# Cost-effectiveness of carbapenem-resistant Enterobacteriaceae (CRE) surveillance in Maryland

**DOI:** 10.1017/ice.2021.361

**Published:** 2022-09

**Authors:** Gary Lin, Katie K. Tseng, Oliver Gatalo, Diego A. Martinez, Jeremiah S. Hinson, Aaron M. Milstone, Scott Levin, Eili Klein, for the CDC Modeling Infectious Diseases in Healthcare Program

**Affiliations:** 1 Center for Disease Dynamics, Economics & Policy, Silver Spring, Maryland, United States; 2 Department of Emergency Medicine, Johns Hopkins University, Baltimore, Maryland, United States; 3 School of Industrial Engineering, Pontificia Universidad Católica de Valparaíso, Valparaíso, Chile; 4 Division of Pediatric Infectious Diseases, Department of Pediatrics, Johns Hopkins University, Baltimore, Maryland, United States; 5 Department of Hospital Epidemiology and Infection Control, The Johns Hopkins Hospital, Baltimore, Maryland, United States; 6 Department of Epidemiology, Johns Hopkins University, Baltimore, Maryland, United States

## Abstract

**Objective:**

We analyzed the efficacy, cost, and cost-effectiveness of predictive decision-support systems based on surveillance interventions to reduce the spread of carbapenem-resistant Enterobacteriaceae (CRE).

**Design:**

We developed a computational model that included patient movement between acute-care hospitals (ACHs), long-term care facilities (LTCFs), and communities to simulate the transmission and epidemiology of CRE. A comparative cost-effectiveness analysis was conducted on several surveillance strategies to detect asymptomatic CRE colonization, which included screening in ICUs at select or all hospitals, a statewide registry, or a combination of hospital screening and a statewide registry.

**Setting:**

We investigated 51 ACHs, 222 LTCFs, and skilled nursing facilities, and 464 ZIP codes in the state of Maryland.

**Patients or participants:**

The model was informed using 2013–2016 patient-mix data from the Maryland Health Services Cost Review Commission. This model included all patients that were admitted to an ACH.

**Results:**

On average, the implementation of a statewide CRE registry reduced annual CRE infections by 6.3% (18.8 cases). Policies of screening in select or all ICUs without a statewide registry had no significant impact on the incidence of CRE infections. Predictive algorithms, which identified any high-risk patient, reduced colonization incidence by an average of 1.2% (3.7 cases) without a registry and 7.0% (20.9 cases) with a registry. Implementation of the registry was estimated to save $572,000 statewide in averted infections per year.

**Conclusions:**

Although hospital-level surveillance provided minimal reductions in CRE infections, regional coordination with a statewide registry of CRE patients reduced infections and was cost-effective.

Healthcare-associated infections (HAIs) pose a significant risk to patient safety, particularly antibiotic-resistant infections, such as carbapenem-resistant Enterobacteriaceae (CRE), which increases the risk of morbidity and mortality.^1^ CRE infections account for an estimated 9,000 HAIs annually in the United States,^2^ with an attributable mortality rate ranging from 26% to 44%.^3^ In Maryland, 4.80 cases of CRE per 100,000 persons were reported between 2012 and 2013, which is significantly higher than an average of 2.93 per 100,000 persons across 7 states.^4^ Although CRE infections can occur in any healthcare facility, including long-term care facilities, most healthcare-associated CRE infections are identified in acute-care hospitals (ACHs).^5^ Identifying patients that are asymptomatically colonized can aid in informing appropriate empiric antimicrobial therapy,^6^ and it allows healthcare providers to institute infection prevention and control (IPC) measures for that patient (e.g., contact precautions, isolation, and CHG bathing) to reduce patient-to-patient transmissions.^7,8^ However, as patients regularly move between healthcare facilities, either through direct transfers or serial admissions to other facilities, reducing the spread of CRE requires a multifaceted approach that tackles both transmission in the hospital and between healthcare settings.

Tracking patients colonized with CRE as they move between healthcare facilities has been shown to better contain the spread of CRE^9^ and to reduce costs associated with identifying colonized patients. For example, in 2013, the Illinois Department of Public Health implemented a voluntary web-based public health registry that tracked patients carrying CRE for 115 ACHs and other facilities.^10^ Simulation-based studies suggested that expansion could be effective at reducing CRE on a regional scale.^11^ However, scaling such a registry requires technological capabilities at each facility and significant financial investment. Given the resource constraints in many healthcare facilities, it is necessary for administrators and policy makers to understand the economics of such a program compared to more traditional surveillance strategies.

In recent years, most ACHs in the United States have converted their medical records to an electronic format and have stored them on an electronic health record (EHR) system. An EHR system can be modified with relative ease and low cost to communicate with other systems to provide or determine information on patients, such as CRE colonization status. Additionally, patients identified to be at risk of colonization at hospital admission can be automatically flagged and IPC interventions can be ordered. Such an implementation in hospitals statewide would potentially be cost-effective, particularly given that most hospitals have IPC programs. To assess the potential benefits, costs, and cost-effectiveness of an automated electronic registry to reduce colonization and infection of CRE, we developed a statewide model of patient movement based on actual patient flows between hospitals in Maryland and compared the impact and cost-effectiveness of interventions with and without a coordinated registry.

## Methods

### Model structure

**Fig. 1.**
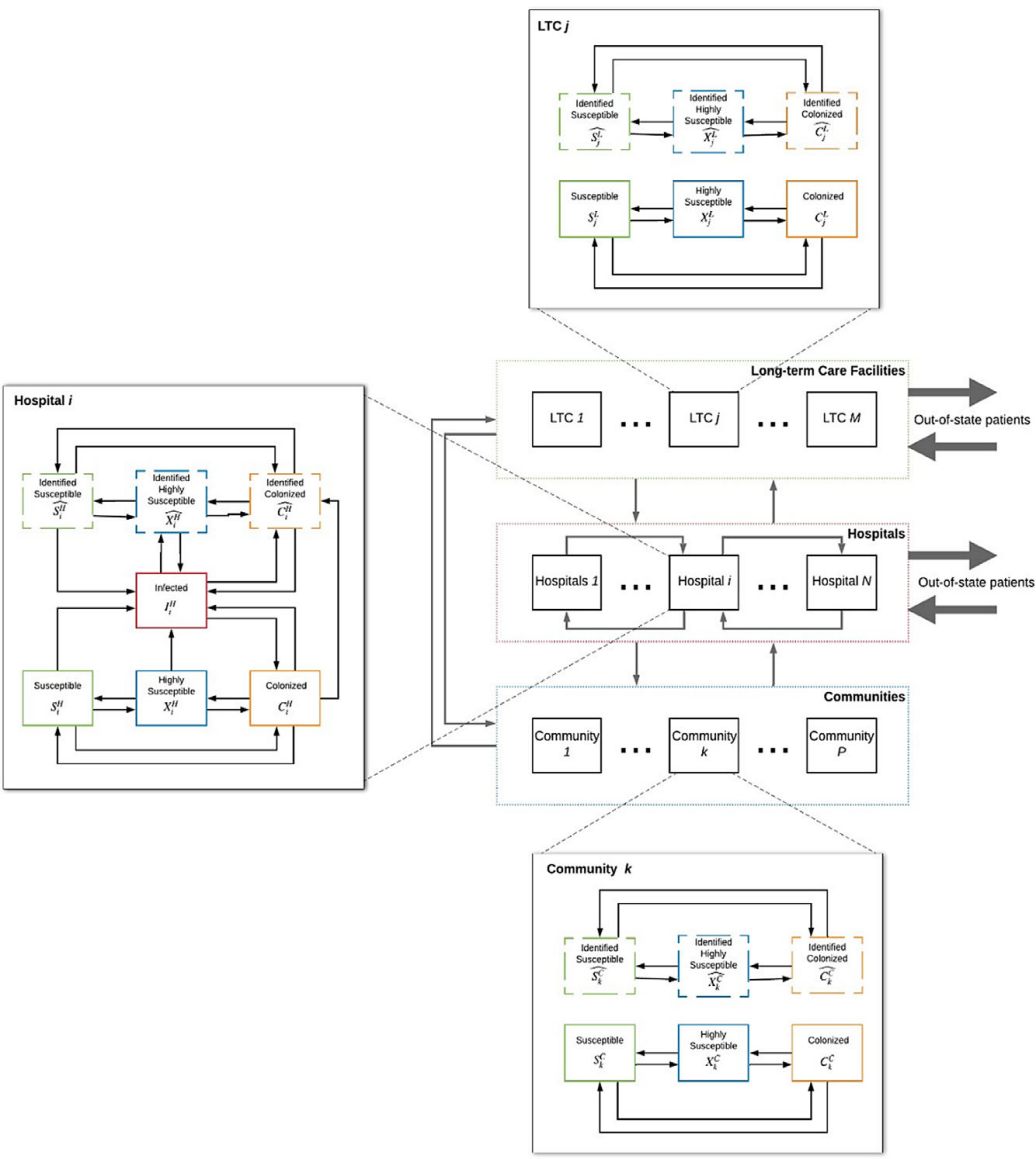
Generalized schematic of the hierarchal metapopulation model. The compartmental state transition for each population is shown in more detail in the supplement. The diagram assumes there is an *M* number of long-term care facilities (LTCFs), *N* acute-care hospitals (ACHs), and *P* communities. The right-middle component in the diagram shows the regional flows of patients between the LTCFs, ACHs, and communities. The compartments for each population shown in the top, left-middle, and bottom components. There are 4 primary compartments in our model susceptible (*S*), higher susceptible (*X*), colonized (*C*), and infected (*I*). For patients that are identified with CRE, they are indicated with a hat, i.e., *Ŝ*, 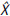 , and *Ĉ*.

We developed a hierarchal metapopulation model^12^ of Maryland to capture the patient population hospital movement network. The model is comprised of a set of ordinary differential equations at 2 scales: (1) within a healthcare facility or community, and (2) movement between facilities and communities. Within this model, patients are assigned to 1 of 4 health states: susceptible (*S*), infected (*I*), or colonized with CRE (*C*), or susceptible but at increased risk of CRE colonization due to antibiotic use (*X*).^13–15^ An additional state for each patient population was included to track patients with an electronic registry, which identified whether a patient has been previously identified as colonized or infected with CRE (Fig. 1 and Supplementary Fig. S1 online). All parameter values that correspond with biological values are listed in Supplementary Table S4 (online).

### Data sources

To model patient flow, we used 2012–2016 patient-mix data from the Maryland Health Services Cost Review Commission (HSCRC), which included patient admissions and transfers for all ACHs in Maryland. Maryland hospitals must report detailed patient visit data to the Health Services Cost Review Commission to obtain reimbursement for care, including demographics, patient home ZIP code, source of admission, discharge location, and whether the patient spent time in an ICU. Data were anonymized; however, a unique patient identifier was assigned to track returning patients to be tracked over time and between hospitals. From these data, we developed a patient movement network that captured relative travel behavior between 51 ACHs (see Supplementary Table S1 online). We further extended this based on admission and discharge data to include movement between ACHs and long-term care facilities (LTCFs) and skilled nursing facilities (SNFs), communities, and patients from out of state. Based on data from Centers for Medicare and Medicaid Services (CMS) and HSCRC, we included 222 LTCFs and SNFs and determined their population size using the number of certified beds.^16^ Communities were defined using the 464 ZIP codes in the state of Maryland, and population sizes were estimated based on data from the US Census Bureau.^17^ Parameters were based on data from the literature as well as hospital-level data (Tables S1 and S2 online).

### Interventions

Our primary aim was to explore the effect of implementing an electronic registry to facilitate targeted IPC implementation at ACH admission for all patients likely to be colonized (Table 1). We assumed patients that were under IPC measures had a 90% reduction in transmission compared to undetected CRE carriers.^18^ We compared the institution of an electronic registry with increased rates of culture-based screening for CRE. To our knowledge, only 1 ACH in Maryland has an established CRE screening program for patients entering select intensive care units (surgical and medical ICUs) and oncology units. Thus, we assumed in our simulated interventions that every other hospital would need to add a program to screen all patients upon admittance to the ICU and weekly thereafter. We considered 2 possible regional screening programs: (1) selective, in which an active CRE surveillance policy was implemented at the 5 ACHs with the highest connectivity to other hospitals, and (2) complete, in which active surveillance was implemented at all 46 ACHs with ICUs in Maryland. We assumed that IPC interventions were implemented for patients with positive results. As an alternative, we considered the implementation of a predictive screening program that assumed that machine-learning algorithms can identify patients at high-risk of CRE colonization at hospital admission with 80% positive predictive value.^19^

**Table 1.** Summary of Scenarios and Interventions^a^

Interventions	Scenarios							
	Baseline	1	2	3	4	5	6	7
Selective screening: The 5 ACHs with the highest flows of patients implement an active surveillance policy to screen all patients admitted to the ICU. Given the network of patient flows between all ACHs, we applied an eigenvector centrality measure to capture the hospital with the most influence on the patient movement network.^42^ The ACHs with the 5 highest eigenvector centrality measures were chosen since previous studies have shown that eigenvector centrality predict higher rates of CRE.^43^ We assumed is a 90% positive predictive value (true positive ÷ detected positive). Patients that were detected were assumed to be on an IPC bundle.	…	Yes	…	…	…	Yes	…	…
Complete screening: Active surveillance of all patients admitted to all ICUs in Maryland, which includes 46 of the 51 ACHs because 5 ACHs did not have an ICU. We assumed a 90% positive predictive detection.	…	…	Yes	…	…	…	Yes	…
Predictive screening: Based on prior work,^44^ we examined the impact of a machine-learning predictive algorithm to identify patients at high-risk of CRE colonization at hospital admission. Pilot programs using predictive algorithms have been implemented at select hospitals in Maryland. We assumed the predictive algorithm had a sensitivity of 80% of detecting colonization and a 90% positive predictive value.	…	…	…	Yes	…	…	…	Yes
Electronic registry: Implementation of a fully electronic statewide registry that would automatically flag all patients with prior colonization or infection with CRE. Patients with positive detection remained detected as they traveled between hospitals.	…	…	…	…	Yes	Yes	Yes	Yes

Note. ACH, acute-care hospital; ICU, intensive care unit; CRE, carbapenem-resistant Enterobacteriaceae; IPC, infection prevention and control. “Yes” in the center grid indicates whether the intervention is implemented for that specific scenario.

a All scenarios (top) in our simulation with corresponding interventions are shown (left).

### Sensitivity analysis

Given the structural assumptions of the model, we analyzed the main factors driving variation in the rates of CRE colonization and incidence using Latin Hypercube Sampling (LHS).^20,21^ Studies have shown that LHS is significantly more efficient than simple random and fractional stratified sampling designs (see Blower and Dowlatabadi^22^) and is a method commonly used to evaluate models within the field of epidemiology.^22–26^ For each scenario, we sampled the parameter space over 300 simulation runs. We sampled outcomes between scenarios using the Welch 2-sample *t* test, and *P* values were reported. Additionally, we conducted a sensitivity analysis regarding the effectiveness of the IPC bundle to reduce transmission (Appendix G online).

### Cost-effective analysis

The average cost for a single CRE surveillance screening was estimated to be $8.65, which is calculated from total costs of swabbing, culturing, conducting organism identification test, antimicrobial susceptibility testing, phenotypic testing, and molecular analysis (see Appendix F online for detailed cost breakdown).

For patients identified as positive, either through culture screening or by the registry, we assumed that the hospital would implement a bundled IPC intervention, which was estimated to cost $639.48 per CRE patient (Appendix F online). Costs were based on a review of the literature and included the cost of placing a patient on contact precautions and the cost of implementing daily chlorhexidine (CHG) bathing for decolonization. Contact precautions were defined as the use of personal protective equipment, including disposable gloves and gowns, which require 1 minute to don and doff per contact. All costs included both material and staffing costs (see Appendix F online). Because any surveillance method trades off between sensitivity and specificity, we assumed that with any method, some false positives resulted in erroneous utilization of the bundled IPC intervention on uncolonized patients, which increased costs with no additional benefit. For the implementation of the electronic registry, costs of modifications to each hospital were based on internal hospital estimates ($10,000 per hospital) so real-time connections to a state server could be made. No additional costs were assumed for the implementation of a predictive screening algorithm.

To determine the cost-effectiveness of each scenario, we calculated the net cost to deliver each intervention scenario, adjusted for the cost savings due to averted infections. The average cost per CRE infection was estimated to be $30,484.^27–29^ The incremental cost-effectiveness ratio (ICER) was calculated by dividing the average difference in net costs between 2 intervention scenarios by the average difference in their effect (eg, number of infections averted by each scenario). Costs and benefits were measured per simulation, and averages and 95% uncertainty Intervals were calculated from the resulting outcomes.

## Results

**Fig. 2.**
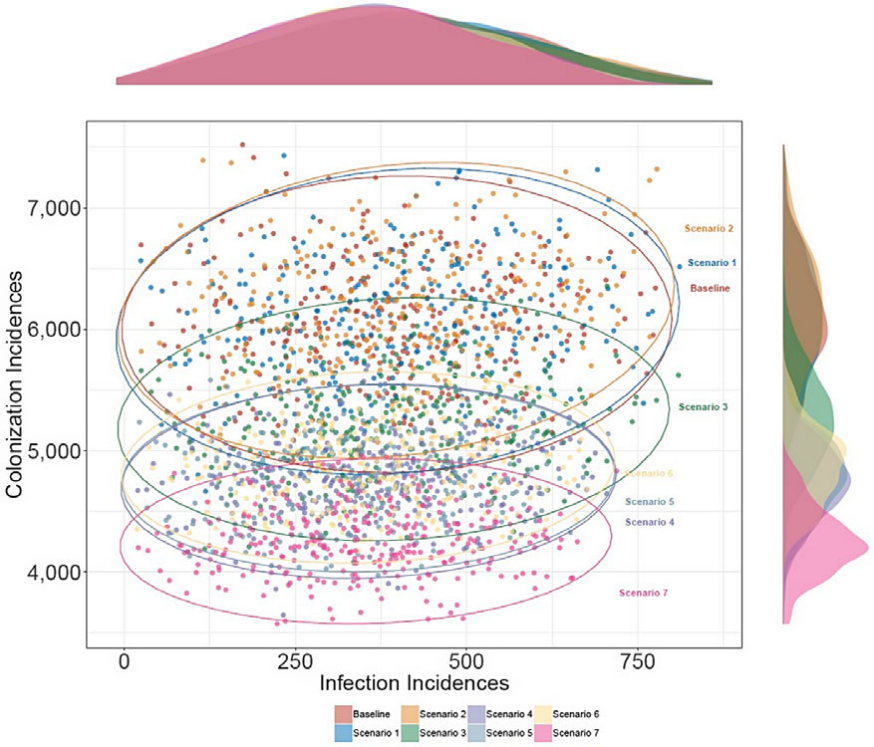
Colonization and infection incidences. Each point on the scatterplot corresponds with the colonization and infection incidence counts for a single simulation of 1 year across all hospitals. The ellipses encircle 95% of simulation runs for each scenario. The probability density of colonization and infection incidences for each scenario are shown on the top and right side of the scatter plot, respectively. There was no statistical difference between scenarios 1–3, which relied only on screening, but the implementation of the electronic registry in scenarios 4–7, reduced the number of colonization events significantly. Given the short time frame of the simulation, the impact on infection was less pronounced but still significant for the registry and would be expected to increase over time since colonization is a major risk factor for infection.

**Fig. 3.**
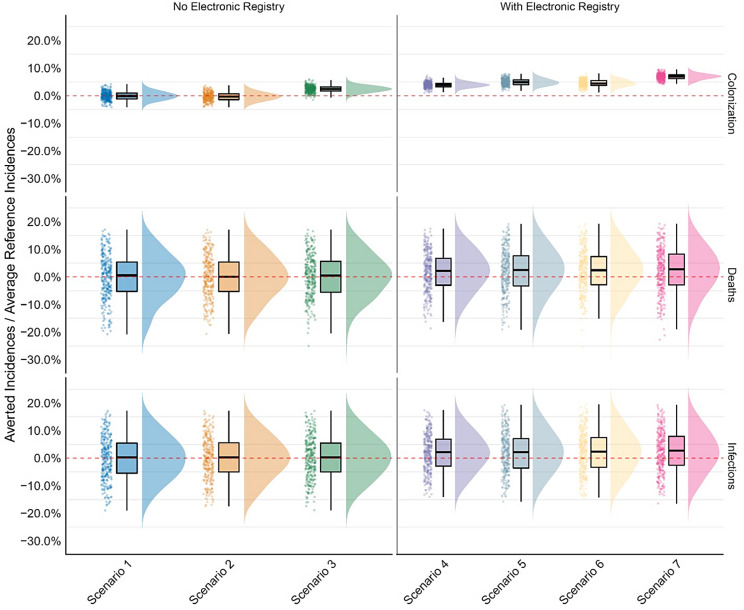
A statewide estimate of net reduction in colonization, deaths, and infections for all acute-care hospitals in Maryland for 1 year for each intervention. The number of averted colonizations, deaths, and infections in scenarios 1, 2, 3, and 4 are compared with the average value in the baseline scenario, while scenarios 5, 6, and 7 are compared with scenario 4. For all measures in each scenario, the raw data, box plot (median, interquartile ranges, 95% uncertainty intervals), and probability density are displayed left to right. Comparison between scenarios with and without an electronic registry shows significant differences in intervention effects on averting colonization, deaths, and infections for interventions that have an electronic registry.

The model outputs with regard to infections and colonization based on the LHS sampling for all scenarios are shown in Figure 2. In the baseline scenario, we fit the model to 298 (95% uncertainty interval [UI], 284–311) CRE infections per annum in Maryland, which equates to 1.20 (95% UI, 1.14–1.25) new infections per 10,000 patient days and 33 CRE-related deaths (95% UI, 32–35) per year. Because only a small proportion of CRE infections are identified, we estimated that there were ˜4,319 new cases of CRE colonization without infection (95% UI, 4,282–4,356) per annum in ACHs, of which only 22 cases (95% UI, 22–23) were identified because the patients were actively observed. The net cost of interventions was $218,000 (95% UI, 209,000–227,000), which includes $12,000 in screening and $207,000 in IPC-related costs (see Appendix F in supplement for cost breakdown). At baseline, we assumed that there were ˜1,298 (standard deviation [SD], 1) screenings each year, including both admission and follow-up swabs, of which 323 (SD, 120; 24.9%) screenings were positive. Of the positive detections, there were 22 detected cases of colonization, 2 false-positive detections, and 298 admitted patients with infections. We compared the effectiveness of interventions (scenarios 1–7) in Figure 3, which shows the net reduction of annual new cases of colonization, deaths, and infections with the baseline scenario.

### Intervention effectiveness on reducing infections and colonization

#### Select, complete, and predictive screening

**Fig. 4.**
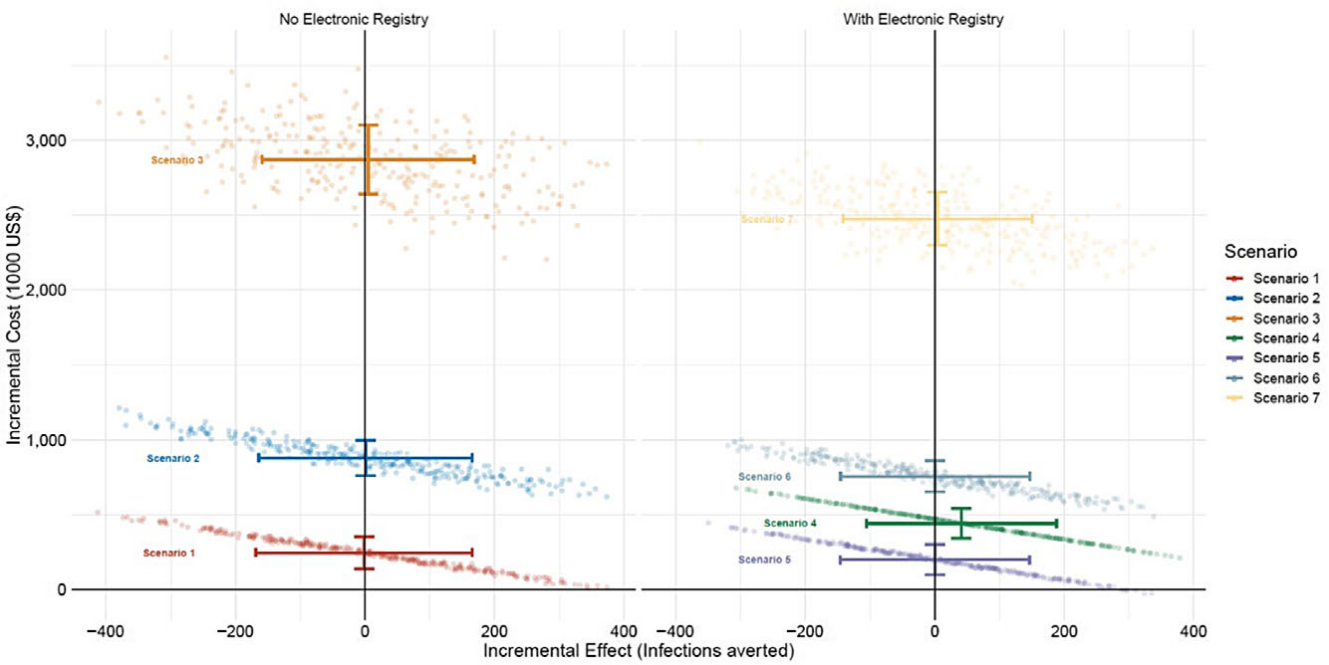
Incremental cost-effectiveness plane for all scenarios. The vertical axis represents the incremental cost, defined as the additional cost compared to the control intervention, and the horizontal axis represents the incremental effect, which is the additional number of infections averted compared to the control intervention. The control intervention for scenarios 1, 2, 3, and 4 is the average cost and averted infections in the baseline scenario; the control interventions for scenarios 5, 6, and 7 is the average cost and averted infections in scenario 4. The vertical and horizontal error bars represent 1 standard deviation range around the mean for incremental cost and effect. Based on the cost-effectiveness, the incremental cost-effectiveness ratio (ICER) is calculated based on mean incremental cost and effectiveness, which indicated that the most cost-effective is scenario 4, with lower incremental cost and higher incremental effect. However, some simulations show instances in which scenarios 1 and 5 have cost-saving and effective outcomes.

Comparing the effectiveness of the screening interventions (Fig. 4), we found that scenario 1, in which surveillance screening was implemented in the 5 ACHs with the highest eigenvector centrality, resulted in a nonstatistically significant change in the annual incidence of CRE infections by 0.7 cases per year (95% UI, −14.7 to 13.2), or 0.2% (95% UI, −4.9% to 4.4%) increase compared to the baseline scenario (*P* = .94) (Fig. 1). Similarly in scenario 2, in which surveillance screening was implemented in all ICU patients, and in scenario 3, in which a predictive algorithm that screens all hospital patients was implemented, we detected no statistically significant change in CRE infections per year (95% UI, −13.3 to 14.1 and 95% UI, −9.8 to 17.2, respectively) compared to the baseline (*P* = .97 and P = .70, respectively). The number of new colonization cases in scenario 1, 4,336 (95% UI, 4,297–4,374), was not significantly different from baseline (*P* = .53); however, new colonization cases varied statistically from the baseline in scenarios 2 and 3, in which colonization cases numbered 4,408 (95% UI, 4,369–4,446) and 3,794 (95% UI, 3,763–3,826), respectively.

#### Electronic registry and combined interventions

The implementation of the electronic registry (scenario 4) reduced annual incident infections by 18.8 cases (95% UI, 5.8–31.7), or 6.3% (95% UI, 2.0%–10.6%; P < .050) compared to the baseline scenario. Complementing the registry by increasing surveillance found that screening, either in highly connected ICUs (scenario 5) or in all ICUs (scenario 6) did not reduce infections significantly compared to the registry alone (P = .96; P = .91). Coupling the electronic registry with a predictive algorithm (scenario 7) further reduced incident infections by 4.1 per year, but this was not a statistically significant change (P = .82). In scenarios 4, 5, 6, and 7, colonization incidences for 1 year was measured at 3,544 (95% UI, 3,516–3,573), 3,556 (95% UI, 3,528–3,585), 3,628 (95% UI, 3,598–3,659), and 3,186 (95% UI, 3,161–3,212) cases, where 34 (95% UI, 33–35; 1%), 114 (95% UI, 113–115; 3%), 458 (95% UI, 454–461; 13%), and 2,388 (95% 2,370–2,406; 75%) cases were detected through screening, respectively. Reduction of colonization cases (detected and undetected) for all scenarios with an electronic registry were statistically significant when compared with the baseline. Scenarios 4, 5, 6, and 7 reduced colonization by 774 (95% UI, 746–803), 763 (95% UI, 734–791), 691 (95% UI, 660–721), and 1132 (95% UI, 1107–1158) cases per year, respectively.

### Cost and cost-effectiveness analysis

Implementation of selective screening (scenario 1) resulted in an average of 10,141 (SD, 5) surveillance swabs per year, and detected an average of 442 (SD, 125; 4.4%) CRE-positive colonized patients, which resulted in an average annual net cost of $393,000 (95% UI, *−*$41,000 to $828,000). Complete screening (scenario 2) resulted in an average of 27,986 (SD, 15) screens per year, detecting 868 (SD, 127; 3.1%) CRE-positive patients, for an annual net cost of $790,000 (Table 2). In scenario 3, the average number of screenings was 19,567 (SD, 169) and produced an average of 3,267 (SD, 244; 16.7%) positive detections with a corresponding annual net cost of $2.15 million (Table 2). The ICER of scenario 1 was *−*$209,000 per infection averted, which is dominated by scenario 2 and 3, which had ICERs of $1.54 million and $552,000 per infection averted, respectively (Fig. 4). Table 3 shows the summarized breakdown of costs associated with each type of intervention.

**Table 2.** Summary of Simulation Output and Cost–Benefit Analysis^a^

Scenario	Patients Screened per Year, No. (UR)	Positive Detections per Year, No. (UR)	Infections Averted Compared to Baseline per Year, No. (UI)	Annual Cost, $1,000s (UI)	Annual Savings, $1,000s (UI)	Annual Net Cost, $1,000s (UI)
Baseline^b^	1,298	323	…	218	…	218
	(1,298–1,298)	(309–336)		(209–226)		(209–226)
Scenario 1^c^	10,141	442	−0.7	371	−22	393
	(10,140–10,141)	(428–457)	(−15–13)	(362–380)	(−448, 403)	(−41–827)
Scenario 2^d^	27,986	868	0.4	797	11	786
	(27,984–27,987)	(854–882)	(−13–14)	(788–806)	(−407–430)	(359–1212)
Scenario 3^e^	19,567	3,267	3.7	2,259	113	2,146
	(19,548–19,586)	(3,240–3,295)	(−10–17)	(2,241–2,276)	(−299–524)	(1,725–2,567)
Scenario 4^f^	1,214	317	18.8	673	572	101
	(1214–1215)	(303–331)	(6–32)	(664–682)	(178–966)	(−302–504)
Scenario 5^g^	9,474	405	19.2	801	586	216
	(9474–9475)	(392–419)	(6–32)	(792–810)	(190–982)	(−189–620)
Scenario 6^h^	26,365	786	19.8	1,191	604	587
	(26,364–26,367)	(773–800)	(7–33)	(1,182–1,200)	(211–996)	(186–988)
Scenario 7^i^	18,232	2,931	20.9	2,492	637	1,855
	(18,216–18,247)	(2,906–2,955)	(8–34)	(2,476–2,508)	(243–1,031)	(1,452–2,258)

Note. UI, uncertainty intervals; ACH, acute-care hospital; CRE, carbapenem-resistant Enterobacteriaceae. All costs are in US$.

a The costs are associated with CRE-related interventions for the entire state of Maryland.

b Baseline scenario: Status quo with no intervention.

c Scenario 1 includes the select screening intervention at 5 ACHs.

d Scenario 2 includes complete screening at all ACHs.

e Scenario 3 includes the predictive algorithm intervention that identifies high-risk patients that should be screened.

f Scenario 4 includes a statewide electronic registry, but otherwise only screening at a single hospital as in the baseline.

g Scenario 5 combines an electronic registry with a select screening at 5 hospitals.

h Scenario 6 combines an electronic registry at all ACHs.

i Scenario 7 combines an electronic registry with a predictive algorithm-based screening strategy.

**Table 3.** Cost Breakdown of Intervention Scenarios in USD per Annum^a^

Scenario	No. of Acute-Care Hospitals Receiving Intervention	Mean No. of Patients Screened per Year	Mean No. of New Positive Detections per Year	Cost of Screening, USD	Cost of EHR Intervention, USD per Annum	Cost of Bundled IPC, USD per Annum
Baseline	1	1,298	323	12,000	0	207,000
Scenario 1	5	10,141	442	88,000	0	283,000
Scenario 2	46	27,986	868	243,000	0	556,000
Scenario 3	46	19,567	3,267	170,000	0	2,090,000
Scenario 4	46	1,214	317	11,000	460,000	203,000
Scenario 5	46	9,474	405	82,000	460,000	260,000
Scenario 6	46	26,365	786	229,000	460,000	503,000
Scenario 7	46	18,232	2,931	158,000	460,000	1,875,000

Note. IPC, infection prevention and control.

a Total cost was calculated as the sum cost of screening, EHR (if implemented), and bundled IPC.

The electronic registry implemented in scenario 4 resulted in an estimated net savings of $101,000 (95% UI, *−*$505,000 to $302,000) per year for ACHs, with $570,000 in savings from fewer infections (Supplementary Appendices online). This was the most cost-effective intervention, with an ICER of $25,000 per averted infection (Fig. 4). Scenario 5 yielded net savings of $216,000 (95% UI, *−*$621,000 to 189,000) with averted infection costs totaling $0.59 million. Scenarios 6 and 7 had net costs of $588,000 (95% UI, $187,000–$989,000) and $1.86 million (95% UI, $1,452,000–$2,259,000), respectively among the scenarios with electronic registries, with savings of $0.60 million and $0.64 million in averted infections. The ICERs of scenarios 5, 6, and 7 were $290,000, $500,000, and $856,000 per averted infection, respectively (Fig. 4).

## Discussion

Combatting the spread of antimicrobial-resistant infections needs to be undertaken regionally. Here, we found that implementation of a statewide registry would lead to considerable cost savings through the prevention of new CRE colonization and infection, mediated exclusively through knowledge sharing across institutions. New case detection through systematic screening yielded no appreciable benefit over a registry alone. Although somewhat surprising, these results are encouraging because registries can be established with relatively minimal investment.

Statewide registries have been shown to reduce MRSA and VRE^30–34^ and could be extended to combat CRE^9^; however, operationalizing coordination can be complex and costly. To date, there is only a limited understanding of the scale needed to cost-effectively reduce CRE infection rates within a region. Illinois has implemented a voluntary registry,^10^ and simulations suggest that expanding this system to all healthcare facilities in the region could reduce all-cause CRE^11^; however, that may not be practicable in all healthcare facilities due to technological and logistical concerns. Most CRE events are asymptomatic colonizations; thus, evaluating the cost-effectiveness of a program to gain state and hospital buy-in, requires understanding the impact on infection incidence over time. Our analysis explored a practical option that could realistically be implemented in most ACHs, an electronic statewide CRE registry that is directly integrated into each hospital’s EHR system. By separately tracking colonization and infection, implementation would reduce the number of CRE infections by ˜18.8 from a current level of 298 CRE infections per annum at a cost of only $25,000 per infection averted ($572,000 in savings).

General recommendations for combatting CRE within a hospital is to proactively screen patients, particularly those at high risk.^35^ We compared different surveillance options, including theoretical introduction of predictive algorithms that could identify patients at high risk of colonization, but these were not particularly cost-effective. Because patients are continually moving between hospitals as well as to other healthcare facilities, and only a fraction of those patients are screened upon hospital entry, most colonized patients are not identified. By adding a mechanism to track patients’ movement between facilities, an electronic registry mitigates the spread of CRE with early detection and action and protects other patients from colonization thus resulting in fewer HAIs.

Our results build on prior work suggesting the importance of regional coordination to combat transmission of CRE,^8,10,36,37^ demonstrating that a practical, feasible system could be cost-effectively implemented across a state. Maryland has some advantages in this respect; it is a small state with a relatively small number of hospitals to coordinate and has already developed statewide reporting mechanisms that could be leveraged to develop such a program. Other areas that are tightly coordinated have shown that these types of strategies can be successful. For example, state- and regional-level coordination in surveillance led to successful control of VRE in the Siouxland region, which crosses the borders of Iowa, Nebraska, and South Dakota.^32–34^ Given the relative advancements in EHR systems in ACHs, implementing an electronic registry is technically feasible. The difficulty lies more in (1) devising systems that do not run afoul of patient privacy concerns and (2) building political will to mandate such a system. Furthermore, an electronic registry improves coordination for many aspects of patient care. Although we only examined CRE transmission, such a system could be leveraged to be useful in other contexts, such as dealing with community-level outbreaks that affect healthcare systems, as well as more mundane patient care issues such as medication compliance.

This study had several limitations. We assumed homogenous mixing rates within each healthcare facility or community. However, HCWs have differential contact patterns in the hospital that drive the transmission of MDROs^36^ and affect the incidence of HAIs. There is also heterogeneity in transmission rates between different LTCFs, ACHs, and communities. The most important assumption of the model is that implementation of IPC interventions, specifically, contact precautions and CHG bathing can limit transmission of CRE. Although controversy regarding the relative effectiveness of these interventions continues,^37^ studies have shown that without IPC interventions CRE rates rise^38,39^; thus, we conservatively assumed that implementation would still allow for 10% transmission; that is, greater efficacy would produce greater gains. However, other studies have suggested a 50% reduction in transmission using an IPC bundle.^40^ Resource limitations at some ACHs might also reduce the effectiveness of an IPC bundle and surveillance measures for that site. A sensitivity analysis was conducted to explore this possibility and included a range of effectiveness from 50% to 90% reduction in transmission. This analysis showed that our results were robust2 and the relative effectiveness of surveillance interventions was consistent across varying IPC effectiveness levels (Appendix G online).

In conclusion, a statewide electronic registry to contain the spread of CRE would be significantly more cost-effective than each hospital conducting their own surveillance and would lead to an effective reduction in HAIs. Additionally, the benefits of investment in a registry would increase year-over-year because the costs associated with implementing the registry are largely front-loaded, while other surveillance strategies require continual investment. We focused on implementation only in ACHs, but wider use by LTCFs and SNFs may be possible, depending on resources to implement the technology and institute interventions.

## Supplementary material

For supplementary material accompanying this paper visit https://doi.org/10.1017/ice.2021.361. click here to view supplementary material
