# Supplementary material for: Cost-effectiveness of carbapenem-resistant Enterobacteriaceae (CRE) surveillance in Maryland
Source: Infect Control Hosp Epidemiol. 2021 Oct 22;43(9):1162–70. doi: 10.1017/ice.2021.361 (PMC9023597; doi:10.1017/ice.2021.361)
Supplement: Supplementary file 1 [file S0899823X21003615sup001.docx]

*Supplementary Appendices*

Supplement to

**Cost-effectiveness of Carbapenem-resistant Enterobacteriaceae (CRE) Surveillance in Maryland**

Gary Lin, Katie K. Tseng, Oliver Gatalo, Diego A. Martinez, Jeremiah S. Hinson, Aaron M. Milstone, Scott Levin, and Eili Klein, for the CDC Modeling Infectious Diseases in Healthcare Program

# Contents

[Contents 1](#_Toc49021736)

[Appendix A Model Populations 2](#_Toc49021737)

[Appendix B Epidemiological Equations 3](#_Toc49021738)

[Appendix C Modeling Patient Movement 11](#_Toc49021739)

[Appendix D Parameters 14](#_Toc49021740)

[Appendix E Calculating the centrality of acute hospitals 17](#_Toc49021741)

[Appendix F Economic evaluation 19](#_Toc49021742)

[Cost calculations 19](#_Toc49021743)

[Cost-effective analysis 20](#_Toc49021744)

# Model Populations

In our model, patients can belong to three types of populations:

- **Hospitals** **–** Any facilities listed as acute-care hospitals (Listed in Table S1)
- **Long Term Care (LTC) Facilities** **–** Any facilities listed as MD/out-of-state skilled nursing facilities or MD/out-of-state long-term care facilities. We assumed patients that are admitted to LTC reside in these facilities.
- **Communities** **–** We assumed that patients that are not a part of any LTC facility belong to a community. We considered each Zip Code Tabulation Area (ZCTA) as a distinct community.
- **Out-of-state –** This included foreign and US-based origins. This population was not modeled explicitly and was simulated as an exogenous in- and outflow of patients in our model.

Table S1. List of Acute Care Hospitals.

| MedStar Franklin Square | Harford Memorial Hospital |
| --- | --- |
| Johns Hopkins | Lifebridge Levindale |
| Johns Hopkins Bayview | Suburban Hospital |
| Lifebridge Sinai Hospital | Prince George’s |
| University of Maryland | MedStar Montgomery General |
| Mercy Medical Center | Western MD Health System |
| Greater Baltimore Medical Center | MedStar Southern Maryland |
| St. Agnes Hospital | Washington Adventist |
| MedStar Union Memorial | Greater Laurel |
| UM Saint Joseph | Doctors Community Hospital |
| MedStar Good Samaritan | UM Shore Medical Center at Easton |
| Lifebridge Northwest Hospital | Mount Washington Pediatric Hospital |
| UM Baltimore Washington Medical Center | Union of Cecil |
| UMM Center Midtown | UM Charles Regional Medical Center |
| Howard General Hospital | Atlantic General |
| MedStar Harbor Hospital | MedStar Saint Mary’s Hospital |
| Anne Arundel Medical Center | Calvert Memorial |
| Bon Secours | UM Shore Medical Center at Dorchester |
| Upper Chesapeake Medical Center | Holy Cross Hospital- Germantown |
| Meritus Health System (Wash. Co.) | Fort Washington |
| Holy Cross Hospital | Adventist Rehabilitation Hospital |
| Carroll County General | UM Shore Medical Center Chestertown |
| UM Rehab & Orthopaedic Institute | Healthsouth Chesapeake Rehab Hospital |
| Frederick Memorial | Garrett County |
| Shady Grove Adventist | McCready |
| Peninsula Regional |  |

# Epidemiological Equations

We define the patient population for each hospital, LTC, and community as $N_{i}^{H}$, $N_{j}^{L}$, and $N_{k}^{C}$. Hospitals are indexed as $i =1,2,\ldots,P$; LTCs are indexed as $j = 1,2,\ldots,Q$; and communities are indexed as $k = 1,2,\ldots,R$.

This model considers the multiscale effects of population dynamics by including the movement of patients to represent regional population dynamics, while the transmission of CRE occurs on a local scale. In hierarchal metapopulation models, homogenous mixing is assumed for each distinct population in order to maintain the parsimony and still capture complex dynamics. By employing compartmental modeling, we were able to define the state variables in Table S2. We assumed that an individual could be in only one of the following four states at a given time: susceptible, highly susceptible, colonized, or infected. Highly susceptible populations include patients that are susceptible but with an increased risk of CRE colonization due to antibiotic use ^1-3^. For the susceptible, highly susceptible, and colonized states, we further distinguish between *identified* and *unidentified* populations which we define as patients that have previously or currently been detected as colonized or infected with CRE. The identified patients are assumed to be limited in transmitting CRE. The purpose of identifying patients is to signal the effects of information asymmetry regarding patient history across healthcare delivery systems. We also assume that patients that were infected with CRE will go directly to the hospital, which are based on the movement rate matrices defined in Appendix C. While some infections are treated in long-term care facilities, we assume that these patients continue to transmit as most LTCs do not have the capability to institute effective contact precautions.

Table S2. List of state variables

|  | Hospital *i* | Long-term care *j* | Community *k* |
| --- | --- | --- | --- |
| Unidentified susceptible | $\boldsymbol{S}_{\boldsymbol{i}}^{\boldsymbol{H}}$ | $\boldsymbol{S}_{\boldsymbol{j}}^{\boldsymbol{L}}$ | $\boldsymbol{S}_{\boldsymbol{k}}^{\boldsymbol{C}}$ |
| Identified susceptible | $\hat{\boldsymbol{S}_{\boldsymbol{i}}^{\boldsymbol{H}}}$ | $\hat{\boldsymbol{S}_{\boldsymbol{j}}^{\boldsymbol{L}}}$ | $\hat{\boldsymbol{S}_{\boldsymbol{k}}^{\boldsymbol{C}}}$ |
| Unidentified, highly susceptible | $\boldsymbol{X}_{\boldsymbol{i}}^{\boldsymbol{H}}$ | $\boldsymbol{X}_{\boldsymbol{j}}^{\boldsymbol{L}}$ | $\boldsymbol{X}_{\boldsymbol{k}}^{\boldsymbol{C}}$ |
| Identified, highly susceptible | $\hat{\boldsymbol{X}_{\boldsymbol{i}}^{\boldsymbol{H}}}$ | $\hat{\boldsymbol{X}_{\boldsymbol{j}}^{\boldsymbol{L}}}$ | $\hat{\boldsymbol{X}_{\boldsymbol{k}}^{\boldsymbol{C}}}$ |
| Unidentified colonized | $\boldsymbol{C}_{\boldsymbol{i}}^{\boldsymbol{H}}$ | $\boldsymbol{C}_{\boldsymbol{j}}^{\boldsymbol{L}}$ | $\boldsymbol{C}_{\boldsymbol{k}}^{\boldsymbol{C}}$ |
| Identified colonized | $\hat{\boldsymbol{C}_{\boldsymbol{i}}^{\boldsymbol{H}}}$ | $\hat{\boldsymbol{C}_{\boldsymbol{j}}^{\boldsymbol{L}}}$ | $\hat{\boldsymbol{C}_{\boldsymbol{k}}^{\boldsymbol{C}}}$ |
| Infected | $\boldsymbol{I}_{\boldsymbol{i}}$ | **--** | **--** |

Table 2 shows the compartment state within each type of population. Figure 1 shows the flows of patients between the state compartments in each population. At the beginning of all scenarios, we assumed that for each population ($N_{i}^{H}$, $N_{j}^{L}$, and $N_{k}^{C}$), 2% were colonized and unidentified ($C_{i}^{H}$, $C_{j}^{L}$, and $C_{k}^{C}$), and the other 98% were susceptible and unidentified ($S_{i}^{H}$, $S_{j}^{L}$, and $S_{k}^{C}$).


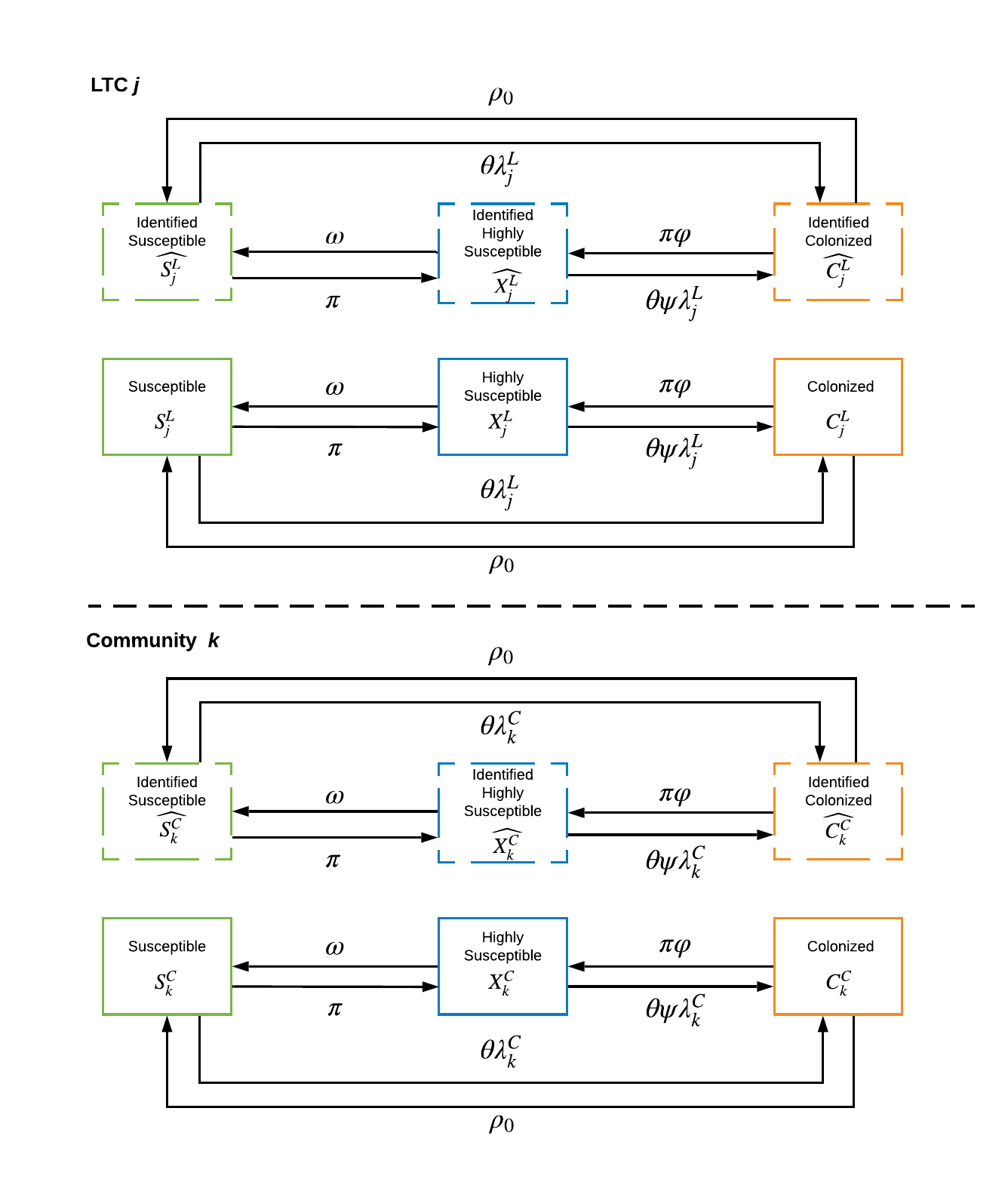

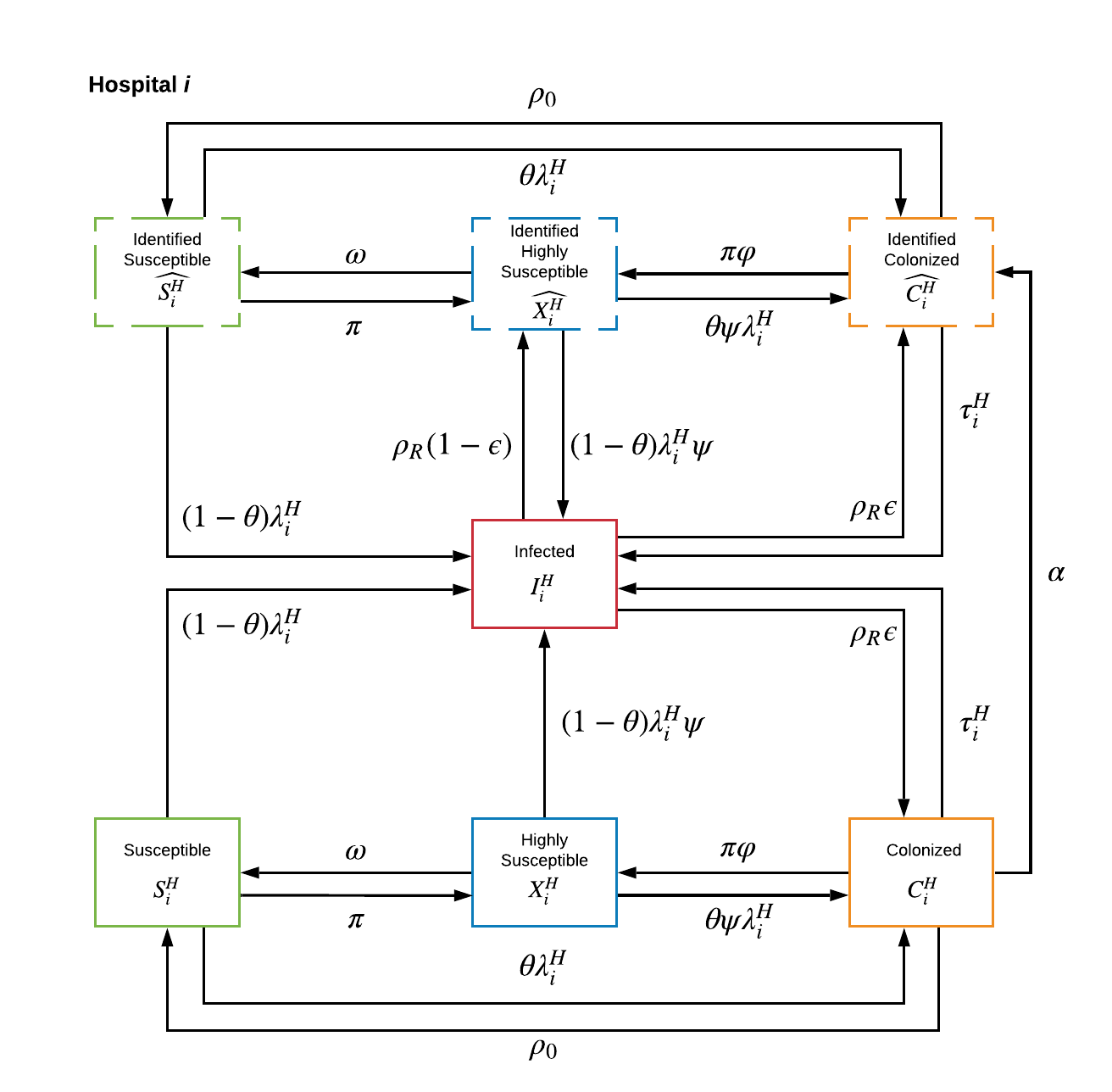


Figure S1. Compartment state flows are shown for acute hospitals (left), long term-care facilities (top right), and community populations (bottom right).

The susceptible patients in our model are defined as the patients that are not colonized, infected by CRE, and at normal susceptibility to CRE (not on antibiotics). We assume that during time $t$, the susceptible patient populations will have the change rate defined in Equations (1) through (6).

| $\frac{d\hat{S_{i}^{H}}}{dt}= \mu\left( \sum_{i^{'}\in D\left( i \right)} \hat{S_{i^{'}}^{H}}\left( t \right)q_{i^{'}i}^{HH}+ \sum_{j=1}^{Q} \hat{S_{j}^{L}}\left( t \right)q_{ji}^{LH} - \sum_{k=1}^{R} \hat{S_{k}^{C}}\left( t \right)q_{ki}^{CH} \right)+ \rho_{0}\hat{C_{i}^{H}}\left( t \right)+ \omega\hat{X_{i}^{H}}\left( t \right)-\hat{S_{i}^{H}}\left( t \right)\left( \lambda_{i}^{H}+\pi_{i}^{H}+q_{i}^{HE}+\sum_{i^{'}\in D\left( i \right)} q_{ii^{'}}^{HH}+\sum_{j=1}^{Q} q_{ij}^{HL}+\sum_{k=1}^{R} q_{ik}^{HC} \right)$ | (1) |
| --- | --- |
| $\frac{dS_{i}^{H}}{dt}=\left( 1-\mu\right)\left( \sum_{i^{'}\in D\left( i \right)} \hat{S_{i^{'}}^{H}}\left( t \right)q_{i^{'}i}^{HH}+ \sum_{j=1}^{Q} \hat{S_{j}^{L}}\left( t \right)q_{ji}^{LH} - \sum_{k=1}^{R} \hat{S_{k}^{C}}\left( t \right)q_{ki}^{CH} \right)+\left( 1-\nu\right)m_{i}^{EH}+\sum_{i^{'}\in D\left( i \right)} S_{i^{'}}^{H}\left( t \right)q_{i^{'}i}^{HH}+ \sum_{j=1}^{Q} S_{j}^{L}\left( t \right)q_{ji}^{LH} + \sum_{k=1}^{R} S_{k}^{C}\left( t \right)q_{ki}^{CH}+ \rho_{0}C_{i}^{H}\left( t \right)+ \omega X_{i}^{H}\left( t \right)-S_{i}^{H}\left( t \right)\left( \lambda_{i}^{H}+\pi_{i}^{H}+q_{i}^{HE}+\sum_{i^{'}\in D\left( i \right)} q_{ii^{'}}^{HH}+\sum_{j=1}^{Q} q_{ij}^{HL}+\sum_{k=1}^{R} q_{ik}^{HC} \right)$ | (2) |
| $\frac{d\hat{S_{j}^{L}}\left( t \right)}{dt}=\mu\left( \sum_{i=1}^{P} \hat{S_{i}^{H}}\left( t \right)q_{ij}^{HL}+\sum_{k=1}^{R} \hat{S_{k}^{C}}\left( t \right)q_{kj}^{CL} \right)+\rho_{0}\hat{C_{j}^{L}}\left( t \right)+\omega\hat{X_{j}^{L}}\left( t \right)-\hat{S_{j}^{L}}\left( t \right)\left( \lambda_{j}^{L}+\pi_{j}^{L}+q_{j}^{LE}+\sum_{i=1}^{P} q_{ji}^{LH}+\sum_{k=1}^{R} q_{jk}^{LC} \right)$ | (3) |
| $\frac{dS_{j}^{L}\left( t \right)}{dt}=\left( 1-\mu\right)\left( \sum_{i=1}^{P} \hat{S_{i}^{H}}\left( t \right)q_{ij}^{HL}+\sum_{k=1}^{R} \hat{S_{k}^{C}}\left( t \right)q_{kj}^{CL} \right)+\left( 1-\nu\right)m_{j}^{EL}+\sum_{i=1}^{P} S_{i}^{H}\left( t \right)q_{ij}^{HL}+\sum_{k=1}^{R} S_{k}^{C}\left( t \right)q_{kj}^{CL}+\rho_{0}C_{j}^{L}\left( t \right)+\omega X_{j}^{L}\left( t \right)-S_{j}^{L}\left( t \right)\left( \lambda_{j}^{L}+\pi_{j}^{L}+q_{j}^{LE}+\sum_{i=1}^{P} q_{ji}^{LH}+\sum_{k=1}^{R} q_{jk}^{LC} \right)$ | (4) |
| $\frac{d\hat{S_{k}^{C}}\left( t \right)}{dt}=\mu\left( \sum_{i=1}^{P} \hat{S_{i}^{H}}\left( t \right)q_{ik}^{HC}+\sum_{j=1}^{Q} \hat{S_{j}^{L}}\left( t \right)q_{jk}^{LC} \right)+\rho_{0}\hat{C_{k}^{C}}\left( t \right)+\omega\hat{X_{k}^{C}}\left( t \right)-\hat{S_{k}^{C}}\left( t \right)\left( \lambda_{k}^{C}+\pi_{k}^{C}+\sum_{i=1}^{P} q_{ki}^{CH}+\sum_{j=1}^{Q} q_{kj}^{CL} \right)$ | (5) |
| $\frac{dS_{k}^{C}\left( t \right)}{dt}=\left( 1-\mu\right)\left( \sum_{i=1}^{P} \hat{S_{i}^{H}}\left( t \right)q_{ik}^{HC}+\sum_{j=1}^{Q} \hat{S_{j}^{L}}\left( t \right)q_{jk}^{LC} \right)+\sum_{i=1}^{P} S_{i}^{H}\left( t \right)q_{ik}^{HC}+\sum_{j=1}^{Q} S_{j}^{L}\left( t \right)q_{jk}^{LC}+\rho_{0}C_{k}^{C}\left( t \right)+\omega X_{k}^{C}\left( t \right)-S_{k}^{C}\left( t \right)\left( \lambda_{k}^{C}+\pi_{k}^{C}+\sum_{i=1}^{P} q_{ki}^{CH}+\sum_{j=1}^{Q} q_{kj}^{CL} \right)$ | (6) |

In the event that an identified patient moves between populations, we assumed that a fraction of patients, defined by *μ*, will stay identified in the $\hat{S_{i}^{H}}$, $\hat{S_{j}^{L}}$, or $\hat{S_{k}^{C}}$ populations, while (1- μ) will become part of the unidentified populations,$S_{i}^{H}$, $S_{j}^{L}$, or $S_{k}^{C}$. The coefficient $\rho_{0}$ is the fraction of colonized patients that spontaneously clear from colonization, $\omega$ is the fraction of higher susceptible patients that are treated with antibiotics returning to normal susceptibility. We also have patient movement rates defined by $q$ (see Appendix C). We assume that normal susceptible patients at hospitals, LTCs, and communities treated with antibiotics become more susceptible to CRE acquisition ^4^, and move out of the normal susceptibility at rates $\pi_{i}^{H}$, $\pi_{j}^{L}$, and $\pi_{k}^{C}$.

The coefficients $\lambda_{i}^{H}$, $\lambda_{j}^{L}$, and $\lambda_{k}^{C}$ are the forces of infection for hospitals, LTCs, and communities, and defined in the following.

| $\lambda_{i}^{H} =\beta^{H}\left( \frac{\sigma^{H}\left( \hat{C_{i}^{H}}\left( t \right)+ I_{i}\left( t \right) \right)+C_{i}^{H}\left( t \right)}{N_{i}^{H}\left( t \right)} \right)$ | (7) |
| --- | --- |
| $\lambda_{j}^{L} =\beta^{L}\left( \frac{\sigma^{L}\hat{C_{i}^{L}}+C_{k}^{L}\left( t \right)}{N_{k}^{L}\left( t \right)} \right)$ | (8) |
| $\lambda_{k}^{C}=\beta^{C}\left( \frac{{\sigma^{C}\hat{C_{i}^{C}}+C}_{k}^{C}\left( t \right)}{N_{k}^{C}(t)} \right)$ | (9) |

where $\beta^{H}$, $\beta^{L}$, and $\beta^{C}$ are the transmission rates for hospitals, LTCs, and communities; $\sigma^{H}$, $\sigma^{L}$, and $\sigma^{C}$ represent the effects of contact precaution in hospitals, LTCs, and communities taken against infected and detected colonized patients on the rate of new infections.

In our model, we included patients at all areas that are at higher susceptibility to antibiotic-resistant bacteria after receiving antibiotics, which are defined as $X_{i}^{H}$, $X_{j}^{L}$, and $X_{k}^{C}$ can be described by the following equation.

| $\frac{d\hat{X_{i}^{H}}\left( t \right)}{dt}=\mu\left( \sum_{i\in D\left( i \right)} \hat{X_{i^{'}}^{H}}\left( t \right)q_{i^{'}i}^{HH}+\sum_{j=1}^{Q} \hat{X_{j}^{L}}\left( t \right)q_{ji}^{LH}+\sum_{k=1}^{R} \hat{X_{k}^{C}}\left( t \right)q_{ki}^{CH} \right)+\pi_{i}^{H}\left( \hat{S_{i}^{H}}\left( t \right)+\varphi\hat{C_{i}^{H}}\left( t \right) \right)+\rho_{R}\left( 1-\epsilon\right)I_{i}\left( t \right)-\hat{X_{i}^{H}}\left( t \right)\left( \psi\lambda_{i}^{H}+\omega+q_{i}^{HE}+\sum_{i^{'}\in D\left( i \right)} q_{ii^{'}}^{HH}+\sum_{j=1}^{Q} q_{ij}^{HL}+\sum_{k=1}^{R} q_{ik}^{HC} \right)$ | (10) |
| --- | --- |
| $\frac{dX_{i}^{H}\left( t \right)}{dt}=\left( 1-\mu\right)\left( \sum_{i\in D\left( i \right)} \hat{X_{i^{'}}^{H}}\left( t \right)q_{i^{'}i}^{HH}+\sum_{j=1}^{Q} \hat{X_{j}^{L}}\left( t \right)q_{ji}^{LH}+\sum_{k=1}^{R} \hat{X_{k}^{C}}\left( t \right)q_{ki}^{CH} \right)+\sum_{i\in D\left( i \right)} X_{i^{'}}^{H}\left( t \right)q_{i^{'}i}^{HH}+\sum_{j=1}^{Q} X_{j}^{L}\left( t \right)q_{ji}^{LH}+\sum_{k=1}^{R} X_{k}^{C}\left( t \right)q_{ki}^{CH}+\pi_{i}^{H}\left( S_{i}^{H}\left( t \right)+\varphi C_{i}^{H}\left( t \right) \right)+\rho_{R}\left( 1-\epsilon\right)I_{i}\left( t \right)-X_{i}\left( t \right)\left( \psi\lambda_{i}^{H}+\omega+q_{i}^{HE}+\sum_{i^{'}\in D\left( i \right)} q_{ii^{'}}^{HH}+\sum_{j=1}^{Q} q_{ij}^{HL}+\sum_{k=1}^{R} q_{ik}^{HC} \right)$ | (11) |
| $\frac{d\hat{X_{j}^{L}}\left( t \right)}{dt}=\mu\left( \sum_{i=1}^{P} \hat{X_{i}^{L}}\left( t \right)q_{ij}^{HL}+\sum_{k=1}^{R} \hat{X_{k}^{C}}\left( t \right)q_{kj}^{CL} \right)+\pi_{j}^{L}\left( \hat{S_{j}^{L}}\left( t \right)+\varphi\hat{C_{j}^{L}}\left( t \right) \right)-\hat{X_{j}^{L}}\left( t \right)\left( \psi\lambda_{j}^{L}+\omega+q_{j}^{LE}+\sum_{i=1}^{P} q_{ji}^{LH}+\sum_{k=1}^{R} q_{jk}^{LC} \right)$ | (12) |
| $\frac{dX_{j}^{L}\left( t \right)}{dt}=\left( 1-\mu\right)\left( \sum_{i=1}^{P} \hat{X_{i}^{L}}\left( t \right)q_{ij}^{HL}+\sum_{k=1}^{R} \hat{X_{k}^{C}}\left( t \right)q_{kj}^{CL} \right)+\sum_{i=1}^{P} X_{i}^{L}\left( t \right)q_{ij}^{HL}+\sum_{k=1}^{R} X_{k}^{C}\left( t \right)q_{kj}^{CL}+\pi_{j}^{L}\left( S_{j}^{L}\left( t \right)+\varphi C_{j}^{L}\left( t \right) \right)-X_{j}^{L}\left( t \right)\left( \psi\lambda_{j}^{L}+\omega+q_{j}^{LE}+\sum_{i=1}^{P} q_{ji}^{LH}+\sum_{k=1}^{R} q_{jk}^{LC} \right)$ | (13) |
| $\frac{d\hat{X_{k}^{C}}\left( t \right)}{dt}=\mu\left( \sum_{i=1}^{P} \hat{X_{i}^{H}}\left( t \right)q_{ik}^{HC}+\sum_{j=1}^{Q} \hat{X_{j}^{L}}\left( t \right)q_{jk}^{LC} \right)+\pi_{k}^{C}\left( \hat{S_{k}^{C}}\left( t \right)+\varphi\hat{C_{k}^{C}}\left( t \right) \right)-\hat{X_{k}^{C}}\left( t \right)\left( \psi\lambda_{k}^{C}+\omega+\sum_{i=1}^{P} q_{ki}^{CH}+\sum_{j=1}^{Q} q_{kj}^{CL} \right)$ | (14) |
| $\frac{dX_{k}^{C}\left( t \right)}{dt}=\left( 1-\mu\right)\left( \sum_{i=1}^{P} \hat{X_{i}^{H}}\left( t \right)q_{ik}^{HC}+\sum_{j=1}^{Q} \hat{X_{j}^{L}}\left( t \right)q_{jk}^{LC} \right)+\sum_{i=1}^{P} X_{i}^{H}\left( t \right)q_{ik}^{HC}+\sum_{j=1}^{Q} X_{j}^{L}\left( t \right)q_{jk}^{LC}+\pi_{k}^{C}\left( S_{k}\left( t \right)+\varphi C_{k}^{C}\left( t \right) \right)-X_{k}^{C}\left( t \right)\left( \psi\lambda_{k}^{C}+\omega+\sum_{i=1}^{P} q_{ki}^{CH}+\sum_{j=1}^{Q} q_{kj}^{CL} \right)$ | (15) |

We assumed that exogenous antibiotic treatment rates, $\pi_{i}^{H}$, $\pi_{k}^{Q}$, and $\pi_{l}^{C}$, varies between each hospital, LTC, and community. The coefficient $\varphi$ is the fraction of colonized patients that take antibiotics who clear the infection. The force of infection among higher susceptible patients are is magnified by the coefficient $\psi$ to capture the increased risk of CRE-acquisition. The flow of treated patients with infections is embedded in Equations (10) and (11), and modified by $\rho_{R}$ is the fraction of infected patients that are treated with antibiotics with $\epsilon$ being the fraction of infected patients that were treated with antibiotic treatment that remain colonized.

We mathematically define the change rate for colonized patients as

| $\frac{d\hat{C_{i}^{H}}\left( t \right)}{dt} = \mu\left( \sum_{i^{'}\in D\left( i \right)} \hat{C_{i}^{H}}\left( t \right) q_{i^{'}i}^{HH}+\sum_{j=1}^{Q} \hat{C_{j}^{L}}\left( t \right) q_{ji}^{LH}+\sum_{k=1}^{R} \hat{C_{k}^{C}}\left( t \right) q_{ki}^{CH} \right)+\kappa\left( \sum_{i^{'}\in D\left( i \right)} C_{i}^{H}\left( t \right) q_{i^{'}i}^{HH}+\sum_{j=1}^{Q} C_{j}^{L}\left( t \right) q_{ji}^{LH}+\sum_{k=1}^{R} C_{k}^{C}\left( t \right) q_{ki}^{CH} \right) +\alpha_{i}C_{i}^{H}(t) +\theta\lambda_{i}^{H} \left( \hat{S_{i}^{H}}\left( t \right) + \psi\hat{X_{i}^{H}}\left( t \right) \right)+\rho_{R}\epsilon I_{i}\left( t \right)-\hat{C_{i}^{H}}\left( t \right)\left( \hat{\tau^{H}}+\pi\varphi+\rho_{0}+q_{i}^{HE}+\sum_{i^{'}\in D\left( i \right)} q_{ii^{'}}^{HH}+\sum_{j=1}^{Q} q_{ij}^{HL}+\sum_{k=1}^{R} q_{ik}^{HC} \right)$ | (16) |
| --- | --- |
| $\frac{dC_{i}^{H}\left( t \right)}{dt} = \left( 1 -\mu\right)\left( \sum_{i^{'}\in D\left( i \right)} \hat{C_{i}^{H}}\left( t \right) q_{i^{'}i}^{HH}+\sum_{j=1}^{Q} \hat{C_{j}^{L}}\left( t \right) q_{ji}^{LH}+\sum_{k=1}^{R} \hat{C_{k}^{C}}\left( t \right) q_{ki}^{CH} \right)+\nu m_{i}^{EH}+\left( 1-\kappa\right)\left( \sum_{i^{'}\in D\left( i \right)} C_{i}^{H}\left( t \right) q_{i^{'}i}^{HH}+\sum_{j=1}^{Q} C_{j}^{L}\left( t \right) q_{ji}^{LH}+\sum_{k=1}^{R} C_{k}^{C}\left( t \right) q_{ki}^{CH} \right) + \theta\lambda_{i}^{H} \left( S_{i}^{H}\left( t \right) + \psi X_{i}^{H}\left( t \right) \right)+\rho_{R}\epsilon I_{i}\left( t \right)-C_{i}^{H}\left( t \right)\left( \tau^{H}+\pi\varphi+\rho_{0}+\alpha_{i}+q_{i}^{HE}+\sum_{i^{'}\in D\left( i \right)} q_{ii^{'}}^{HH}+\sum_{j=1}^{Q} q_{ij}^{HL}+\sum_{k=1}^{R} q_{ik}^{HC} \right)$ | (17) |
| $\frac{d\hat{C_{j}^{L}}\left( t \right)}{dt}=\mu\left( \sum_{i=1}^{P} \hat{C_{i}^{H}}\left( t \right)q_{ij}^{HL}+\sum_{k=1}^{R} \hat{C_{k}^{C}}\left( t \right) q_{kj}^{CL} \right)+\theta\lambda_{j}^{L}\left( S_{j}^{L}\left( t \right)+\psi X_{j}^{L}\left( t \right) \right)-\hat{C_{j}^{L}}\left( t \right)\left( \tau^{L}+\pi\varphi+\rho_{0}+q_{j}^{LE}+\sum_{i=1}^{P} q_{ji}^{LH}+\sum_{k=1}^{R} q_{jk}^{LC} \right)$ | (18) |
| $\frac{dC_{j}^{L}\left( t \right)}{dt}=\left( 1-\mu\right)\left( \sum_{i=1}^{P} \hat{C_{i}^{H}}\left( t \right)q_{ij}^{HL}+\sum_{k=1}^{R} \hat{C_{k}^{C}}\left( t \right) q_{kj}^{CL} \right)+\nu m_{j}^{EL}+\sum_{i=1}^{P} C_{i}^{H}\left( t \right)q_{ij}^{HL}+\sum_{k=1}^{R} C_{k}^{C}\left( t \right) q_{kj}^{CL}+\theta\lambda_{j}^{L}\left( S_{j}^{L}\left( t \right)+\psi X_{j}^{L}\left( t \right) \right)-C_{j}^{L}\left( t \right)\left( \tau^{L}+\pi\varphi+\rho_{0}+q_{j}^{LE}+\sum_{i=1}^{P} q_{ji}^{LH}+\sum_{k=1}^{R} q_{jk}^{LC} \right)$ | (19) |
| $\frac{d\hat{C_{k}^{C}}\left( t \right)}{dt}=\mu\left( \sum_{i=1}^{P} \hat{C_{i}^{H}}\left( t \right)q_{ik}^{HC}+\sum_{j=1}^{Q} \hat{C_{j}^{L}}\left( t \right) q_{jk}^{LC} \right)+\theta\lambda_{k}^{C}\left( \hat{S_{k}^{C}}\left( t \right)+\psi\hat{X_{k}^{C}}\left( t \right) \right)-\hat{C_{k}^{C}}\left( t \right)\left( \tau^{C}+\pi\varphi+\rho_{0}+\sum_{i=1}^{P} q_{ki}^{CH}+\sum_{j=1}^{Q} q_{kj}^{CL} \right)$ | (20) |
| $\frac{dC_{k}^{C}\left( t \right)}{dt}=(1-\mu)\left( \sum_{i=1}^{P} \hat{C_{i}^{H}}\left( t \right)q_{ik}^{HC}+\sum_{j=1}^{Q} \hat{C_{j}^{L}}\left( t \right) q_{jk}^{LC} \right)+\sum_{i=1}^{P} C_{i}^{H}\left( t \right)q_{ik}^{HC}+\sum_{j=1}^{Q} C_{j}^{L}\left( t \right) q_{jk}^{LC}+\theta\lambda_{k}^{C}\left( S_{k}^{C}\left( t \right)+\psi X_{k}^{C}\left( t \right) \right)-C_{k}^{C}\left( t \right)\left( \tau^{C}+\pi\varphi+\rho_{0}+\sum_{i=1}^{P} q_{ki}^{CH}+\sum_{j=1}^{Q} q_{kj}^{CL} \right)$ | (21) |

The coefficient *θ* is the fraction of patients that become colonized and not infected through transmission, $\tau$ is the fraction of colonized patients that become infected. The rate at which colonized patients are detected is defined by $\alpha_{i}$. As mentioned before, we assume that detected colonized patients are only present in hospitals.

Finally, we have the rate at which patients receive an infection. In our model, patients residing in LTCs and communities that transition into the infected state will move to the hospital, which is based on the movement adjacency matrix described in Appendix C.

| $\frac{dI_{i}\left( t \right)}{dt}=\sum_{j=1}^{Q} \left( \tau^{L}\left( \hat{C_{j}^{L}}\left( t \right)+C_{j}^{L}\left( t \right) \right)+\lambda_{j}^{L}\left( 1-\theta\right)\left( \hat{S_{j}^{L}}(t)+S_{j}^{L}(t)+\psi\left( \hat{X_{j}^{L}}(t)+X_{j}^{L}(t) \right) \right) \right)r_{ji}^{LH}+\sum_{k=1}^{R} \left( \tau^{C}\left( \hat{C_{k}^{C}}\left( t \right)+C_{k}^{C}\left( t \right) \right)r_{ki}^{CH}+\lambda_{k}^{C}\left( 1-\theta\right)\left( \hat{S_{k}^{C}}(t)+S_{k}^{C}(t)+\psi\left( \hat{X_{k}^{C}}(t)+X_{k}^{C}(t) \right) \right) \right)+\hat{\tau^{H}}\hat{C_{i}^{H}}\left( t \right)+\tau^{H}C_{i}^{H}\left( t \right)+\lambda_{i}^{H}\left( 1-\theta\right)\left( S_{i}^{H}+\psi X_{i}^{H} \right)-I_{i}\left( t \right)\left( \rho_{R}+\gamma\right)$ | (22) |
| --- | --- |

# Modeling Patient Movement

For this model, there are seven flows of patient movements. The first type included patients that travel from their homes (communities) to the hospital. These only included patients that are admitted to the inpatient wards and stay at the hospital for a couple of days and go back to their homes. The second type of movement included patients that arrive at the hospital from LTCs that stay in the inpatient units and discharged from the hospital. Lastly, we have patients that were transferred and admitted between hospitals, which are flagged as transferred. Figure S2 shows the patient flows in our model.

Patient movement can be generally be described as a movement flow matrix, *M*, where each element $m_{ii^{'}}$ represents the number of patients that traveled from origin $i$ to destination $i'$. The movement flow matrix can also be thought of as an adjacency matrix for a network.

Given that we have $P$ hospitals, the movement flow matrix that characterizes inter-hospital transfers is a $P\times P$ matrix, $M^{HH}$. Flows between LTCs and hospitals are characterized as $M^{LH}$, a $Q\times P$ matrix where each row index represents the origin index, and the column index represents the destination hospital. We also have a reverse movement flow matrix $M^{HL}$ with dimensions $P\times Q$. Finally, the flows between communities and hospitals are captured by $M^{CH}$ and $M^{HC}$ which have dimensions $R\times P$ and $P\times R$. For the purpose of generality, we assumed patient flows were not reversible such that the number of patients that move from origin $i$ to destination $i'$ is not equal to patients moving from origin $i'$ to destination $i$, *i.e.*, $M^{HC}\neq M^{CH}$. In all, we have five movement flow matrices. In order to consider patients that travel to Maryland for healthcare from out-of-state, we added an exogenous inflow, $m_{i}^{EH}$ and $m_{j}^{EL}$ for all acute-care hospital and LTCs. The inclusion criteria based on data for determining the movement magnitudes in Appendix D.


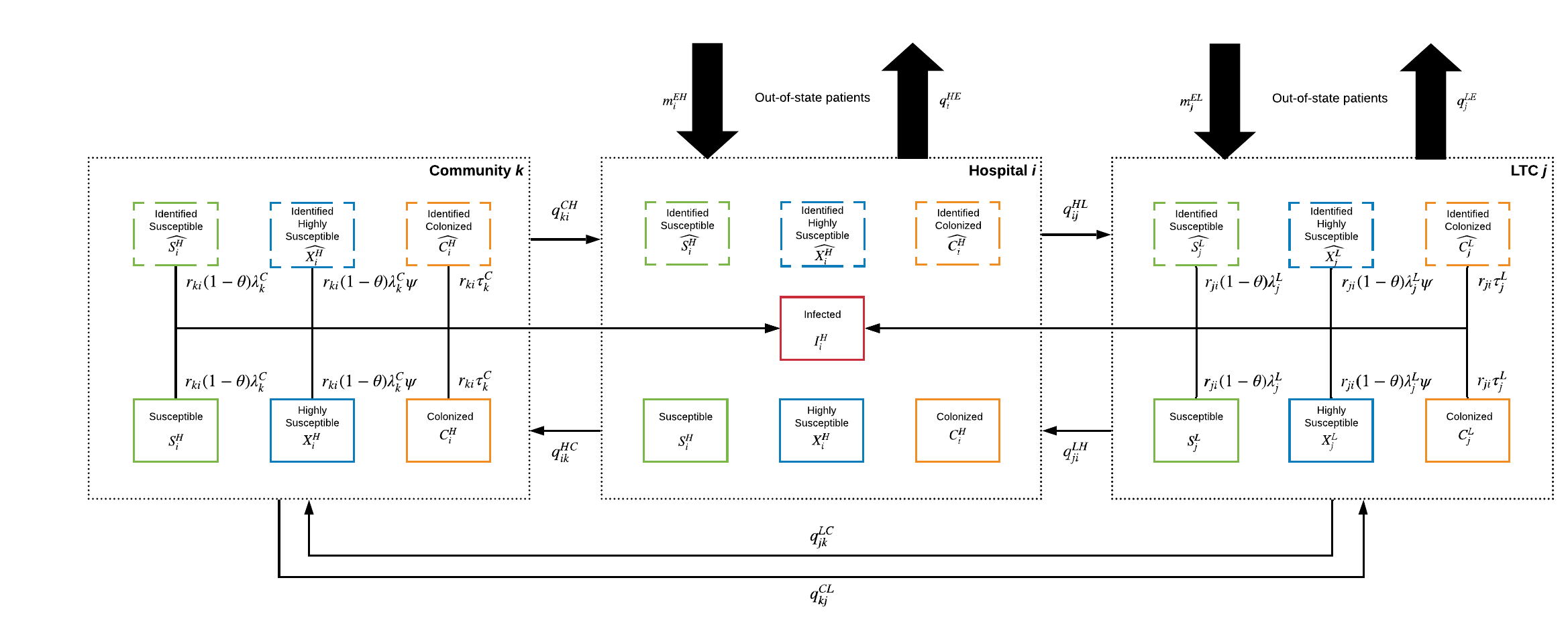


Figure S2. Patient movement flows between Hospitals, LTCs, and Communities. We assumed in our model that colonized people who become infected in communities and LTCs directly go to the hospital without mixing with the LTC and community population.

Each movement flow matrix was converted to movement rates by normalizing the rows by the origin population. In other words, given the patient flow $m_{ij}\in M$ from origin $i$ to destination $j$, we define the movement rate, $q_{ij}$, as

| $q_{ij}=\frac{m_{ij}}{N_{i}}$ | (23) |
| --- | --- |

We have seven directional movement coefficients within Maryland that are defined by Equation (23): $q_{ii^{'}}^{HH}$, LTC/hospital, $q_{ij}^{HL}$ and $q_{ji}^{LH}$, community/hospital movement, $q_{ik}^{HC}$ and $q_{ki}^{CH}$. We also have two outflow rates for patients leaving Maryland, $q_{i}^{HE}$ and $q_{j}^{LE}$.

Similarly, we define a transition rate that represents the probability of an agent moving to destination *j*. This is formally defined as

| $r_{ij}=\frac{m_{ij}}{\sum_{i} m_{ij}}$ | (24) |
| --- | --- |

Eq. (24) provides us values for $r_{ji}^{LH}$ and $r_{ki}^{CH}$. We can now define the equations of motions for patient population size for hospitals, LTC, and communities. We assume that the total population for each hospital is $N_{i}^{H}=S_{i}^{H}+\hat{S_{i}^{H}}+X_{i}^{H}+\hat{X_{i}^{H}}+C_{i}^{H}+\hat{C_{i}^{H}}+I_{i}$. From this identity, we can formulate the following differential equation.

| $\frac{dN_{i}^{H}}{dt}=m_{i}^{EH}+\sum_{i^{'}\in D\left( i \right)} \left( N_{i^{'}}^{H}\left( t \right)-I_{i^{'}}\left( t \right) \right)q_{i^{'}i}^{HH}+\sum_{j=1}^{Q} N_{j}^{L}\left( t \right)q_{ji}^{LH}+\sum_{k=1}^{R} N_{k}^{C}\left( t \right)q_{ki}^{CH}-m_{i}^{HE}-\left( N_{i}^{H}\left( t \right)-I_{i}\left( t \right) \right)\sum_{i^{'}\in D\left( i \right)} q_{ii^{'}}^{HH}-N_{i}^{H}\left( t \right)\sum_{j=1}^{Q} q_{ij}^{HL}- N_{i}^{H}\left( t \right)\sum_{k=1}^{R} q_{ik}^{HC}+\sum_{j=1}^{Q} \left( \tau^{L}\left( \hat{C_{j}^{L}}\left( t \right)+C_{j}^{L}\left( t \right) \right)+\lambda_{j}^{L}\left( 1-\theta\right)\left( \hat{S_{j}^{L}}(t)+S_{j}^{L}(t)+\psi\left( \hat{X_{j}^{L}}(t)+X_{j}^{L}(t) \right) \right) \right)r_{ji}^{LH}+\sum_{k=1}^{R} \left( \tau^{C}\left( \hat{C_{k}^{C}}\left( t \right)+C_{k}^{C}\left( t \right) \right)+\lambda_{k}^{C}\left( 1-\theta\right)\left( \hat{S_{k}^{C}}(t)+S_{k}^{C}(t)+\psi\left( \hat{X_{k}^{C}}(t)+X_{k}^{C}(t) \right) \right) \right)r_{ki}^{CH}$ | (25) |
| --- | --- |

where $D(i) = \{1,2,\ldots,P\}$. We assumed that all patients who become infected are transferred immediately to the hospital, which is based on movement rates. In order to account for infection-related admittances, we included the flows from colonized patients and susceptible that become infected in LTCs and communities in Equation (25) represented in red.

We define the total population of LTC and communities as $N_{j}^{L}=S_{j}^{L}+\hat{S_{j}^{L}}+X_{j}^{L}+\hat{X_{j}^{L}}+C_{j}^{L}+\hat{C_{j}^{L}}$ and $N_{k}^{C}=S_{k}^{C}+\hat{S_{k}^{C}}+X_{k}^{C}+\hat{X_{k}^{C}}+C_{k}^{C}+\hat{C_{k}^{C}}$. We defined the change in population for LTC and communities as

| $\frac{dN_{j}^{L}}{dt}=\sum_{i=1}^{P} N_{i}^{H}\left( t \right)q_{ij}^{HL}+\sum_{k=1}^{R} N_{k}^{C}(t)q_{kj}^{CL}-N_{j}^{L}\left( t \right)\sum_{j=1}^{P} q_{ji}^{LH}-N_{k}^{C}(t)\sum_{k=1}^{R} q_{jk}^{LC}-\left( \tau^{L}\left( \hat{C_{j}^{L}}\left( t \right)+C_{j}^{L}\left( t \right) \right)+\lambda_{j}^{L}\left( 1-\theta\right)\left( \hat{S_{j}^{L}}\left( t \right)+S_{j}^{L}\left( t \right)+\psi\left( {\hat{X_{j}^{L}}\left( t \right)+X}_{j}^{L}\left( t \right) \right) \right) \right)$ | (26) |
| --- | --- |
| $\frac{\text{d}N_{k}^{C}\left( t \right)}{\text{d}t}=\sum_{i=1}^{R} N_{i}^{H}\left( t \right)q_{ik}^{HC}+\sum_{j=1}^{Q} N_{j}^{L}(t)q_{jk}^{LC}-N_{k}^{C}\left( t \right)\sum_{k=1}^{R} q_{ki}^{CH}-N_{j}^{L}(t)\sum_{j=1}^{Q} q_{kj}^{CL}-\left( \tau^{C}\left( \hat{C_{k}^{C}}\left( t \right)+C_{k}^{C}\left( t \right) \right)+\lambda_{k}^{C}\left( 1-\theta\right)\left( \hat{S_{k}^{C}}(t)+S_{k}^{C}(t)+\psi\left( {\hat{X_{k}^{C}}\left( t \right)+X}_{k}^{C}(t) \right) \right) \right)$ | (27) |

# Parameters

Table 3 shows the input parameters are adjusted based on interventions. For acute care hospitals (ACH) implementing screening surveillance in their intensive care unit (ICU), i.e., Scenarios 1, 2, 4, 5, and 6, we calculated $\kappa_{i}$ based on the following formula.

$$\kappa_{i}=\left( Percentage of patients entering the ICU \right)\times\left( 90\% Detection Sensitivity \right)$$

For hospitals that do not implement screening interventions, we assume $\kappa_{i}=0$. In Scenarios 3 and 7, we assumed that hospitals use a predictive algorithm to screen all patients being admitted into the inpatient wards with an 80 percent sensitivity rate. Hence, $\kappa_{i}=0.8$ for all hospitals.

For all scenarios, if the hospital is implementing active surveillance on patients that are already admitted, the parameter $\alpha_{i}$ takes the value

$$\alpha_{i}=\left( Percentage of patients in the ICU \right)\times\left( 90\% Detection Sensitivity \right)\times\left( \frac{1}{7} Proportion tested per day \right)$$

Otherwise, $\alpha_{i}$ is zero.

Based on current practice, we assumed that $\kappa_{i}$ and $\alpha_{i}$ in the baseline scenario are reduced by half since only half the ICUs in this hospital implement screening.

| **Table S3. List of adjusted parameters and values for each scenario** | | | |
| --- | --- | --- | --- |
| *Parameter* | *Description* | *Value* | *Units* |
| $\boldsymbol{\kappa}_{\boldsymbol{i}}$ | Proportion of ICU patients at hospital *i* that are detected as colonized upon admission | Baseline, Scenarios 4: one hospital has a nonzero value  Scenarios 1 and 5: five hospital have a nonzero value  Scenarios 2, 3, 6, and 7: 46 hospital have a nonzero value | Fraction |
| $\boldsymbol{\alpha}_{\boldsymbol{i}}$ | Proportion of ICU patients at hospital *i* detected as colonized | Baseline, Scenarios 4: one hospital has a nonzero value  Scenarios 1 and 5: five hospital have a nonzero value  Scenarios 2, 3, 6, and 7: 46 hospital have a nonzero value | Fraction |
| $\boldsymbol{\mu}$ | Data registry coordination parameter | Baseline, Scenarios 1, 2, 3: set to 0  Scenarios 4, 5, 6, and 7: set to 1 | Fraction |

The fixed parameter values that we used to initialize our simulations are listed in Table S4.

| **Table S4. List of parameters and values** | | | | | |
| --- | --- | --- | --- | --- | --- |
| *Parameter* | *Description* | *Mode*  *(Low, High)* | *Units per day* | *Ref.* |  |
| $\boldsymbol{q}_{\boldsymbol{i}\boldsymbol{i}^{\boldsymbol{'}}}^{\boldsymbol{HH}}$ | Rate of patients transferred from hospital *i* and hospital *i'* relative to the origin population | Fixed Matrix | Per person | HSCRC* |  |
| $\boldsymbol{q}_{\boldsymbol{ij}}^{\boldsymbol{HL}}$,$\boldsymbol{q}_{\boldsymbol{ji}}^{\boldsymbol{LH}}$ | Rate of patients transferred between hospital *i* and LTC *j* relative to the origin population | Fixed Matrix | Per person | HSCRC* |  |
| $\boldsymbol{q}_{\boldsymbol{ik}}^{\boldsymbol{HC}}$,$\boldsymbol{q}_{\boldsymbol{ki}}^{\boldsymbol{CH}}$ | Rate of patients transferred between hospital *i* and community *k* relative to the origin population | Fixed Matrix | Per person | HSCRC* |  |
| $\boldsymbol{q}_{\boldsymbol{jk}}^{\boldsymbol{LC}}$,$\boldsymbol{q}_{\boldsymbol{kj}}^{\boldsymbol{CL}}$ | Rate of patients transferred between LTC *j* and community *k* relative to the origin population | Fixed Matrix | Per person | HSCRC* |  |
| $\boldsymbol{q}_{\boldsymbol{i}}^{\boldsymbol{HE}}$ | Rate of patients that leave Maryland relative from hospital *i* population | Fixed Matrix | Per person | HSCRC* |  |
| $\boldsymbol{q}_{\boldsymbol{j}}^{\boldsymbol{LE}}$ | Rate of patients that leave Maryland relative to LTC *j* population | Fixed Matrix | Per person | HSCRC* |  |
| $\boldsymbol{r}_{\boldsymbol{ji}}^{\boldsymbol{LH}}$ | Transition rate of patients transferred from LTC *j* to hospital *i* relative to the moving population | Fixed Matrix | Per person | HSCRC* |  |
| $\boldsymbol{r}_{\boldsymbol{ki}}^{\boldsymbol{CH}}$ | Transition rate of patients transferred from community *k* to hospital *i* relative to the moving population | Fixed Matrix | Per person | HSCRC* |  |
| $\boldsymbol{m}_{\boldsymbol{i}}^{\boldsymbol{EH}}$ | Number of out-of-state patients that enter from hospital *i* | Fixed Matrix | People | HSCRC* |  |
| $\boldsymbol{m}_{\boldsymbol{j}}^{\boldsymbol{EL}}$ | Number of out-of-state patients that enter LTC *j* | Fixed Matrix | People | HSCRC* |  |
| $\boldsymbol{\gamma}$ | Infection mortality rate | 0.02  (0.016, 0.024) | Per colonized person | ^5^ |  |
| $\boldsymbol{\sigma}$ | IPC bundle reduction in transmission multiplier | 0.1  (0.08, 0.12) | Fraction | ^6^ |  |
| $\boldsymbol{\varphi}$ | Probability of a colonized patients that receive antibiotic treatment completely clears colonization | 0.0153  (0.0122, 0.0184) | Fraction | ^7^ |  |
| $\boldsymbol{\omega}$ | Rate of treated patients returning to normal susceptibility after treatment | 1 / 5.2  (0.385, 0.128) | Per person | Author Estimate |  |
| $\boldsymbol{\rho}_{\boldsymbol{0}}$ | Probability of colonized patients spontaneously cleared of CRE colonization | 1 / 180  (0.0044, 0.0067) | Per person | ^8^ |  |
| $\boldsymbol{\pi}_{\boldsymbol{i}}^{\boldsymbol{H}}$ | Antibiotic treatment rate at hospital $i$ | 0.2  (0.16, 0.24) | Per person | ^9^ |  |
| $\boldsymbol{\pi}_{\boldsymbol{j}}^{\boldsymbol{L}}$ | Antibiotic treatment rate at LTC $j$ | 0.1  (0.08, 0.12) | Per person | Author Estimate |  |
| $\boldsymbol{\pi}_{\boldsymbol{k}}^{\boldsymbol{C}}$ | Antibiotic treatment rate at community $k$ | 0.01  (0.08, 0.12) | Per person | Author Estimate |  |
| $\boldsymbol{\rho}_{\boldsymbol{R}}$ | Proportion of infected patients that are treated with antibiotics | 1 / 6.2  (0.129, 0.194) | Per person | Author Estimate |  |
| $\boldsymbol{\epsilon}$ | Proportion of infected patients that treated with antibiotic treatment that remain colonized | 0.87  (0.696, 1.044) | Per person | ^10, 11^ |  |
| $\boldsymbol{\psi}$ | Increase in susceptibility of antibiotic treated patients to acquire a colonization or infection | 1.5  (1.2, 1.8) | Fraction | ^12, 13^ |  |
| $\boldsymbol{\theta}$ | Proportion of non-carrier patients that are exposed and become colonized without an infection | 0.995  (0.796, 1.194) | Per person | ^14^ |  |
| $\boldsymbol{\tau}_{\boldsymbol{i}}^{\boldsymbol{H}}$ | Proportion of undetected colonized patients that become infected at hospital $i$ | 5×10^-13^  (4×10^-13^, 6×10^-13^) | Per person | Calibrated |  |
| $\hat{\boldsymbol{\tau}_{\boldsymbol{i}}^{\boldsymbol{H}}}$ | Proportion of detected colonized patients that become infected at hospital $i$ | 1×10^-13^  (8×10^-14^, 1.2×10^-13^) | Per person | Calibrated |  |
| $\boldsymbol{\tau}_{\boldsymbol{j}}^{\boldsymbol{L}}$ | Proportion of detected and undetected colonized patients that become infected at LTC $j$ | 1×10^-13^  (8×10^-14^, 1.2×10^-13^) | Per person | Calibrated |  |
| $\boldsymbol{\tau}_{\boldsymbol{k}}^{\boldsymbol{C}}$ | Proportion of detected and undetected colonized patients that become infected at community $k$ | 1×10^-13^  (8×10^-14^, 1.2×10^-13^) | Per person | Calibrated |  |
| $\boldsymbol{\beta}_{\boldsymbol{i}}^{\boldsymbol{H}}$ | Transmission rate for hospital $i$ | 0.03  (0.024, 0.036) | Probability of transmission | Author Estimate |  |
| $\boldsymbol{\beta}_{j}^{\boldsymbol{L}}$ | Transmission rate for LTC $j$ | 0.03  (0.024, 0.036) | Probability of transmission | Author Estimate |  |
| $\boldsymbol{\beta}_{\boldsymbol{k}}^{\boldsymbol{C}}$ | Transmission rate for community $k$ | 0.001  (0.0008, 0.0012) | Probability of transmission | Author Estimate |  |
| $\boldsymbol{\nu}$ | Ratio of out-of-state patients that are colonized. | 0.02  (0.02, 0.02) | Fraction | Initialized |  |
| * Values were estimated using the Maryland Health Services Cost Review Commission (HSCRC) patient mix data | | | | | |

# Calculating the centrality of acute hospitals

We conducted a network analysis where a bipartite network (two-modes) of acute-care hospitals and communities are defined as nodes, and the links defined by patient movement. Based on constructed network, three centrality measures were calculated using the *Igraph* package in R: Betweenness centrality, degree centrality, and eigenvector centrality. Eigencentrality was determined to be the most appropriate measure since capture the hospital with the most influence on the patient movement network ^15^. The top five hospitals with the highest eigenvector centrality were chosen to partake in screening measures for the *selective screening* intervention in Scenarios 1 and 5.

Table S5. List of acute-care hospitals and their centrality measure based on patient-movement network between ACHs and communities. The top five ACHs with the highest eigenvector centrality are highlighted in yellow.

| *Provider Names* | *Betweenness Centrality* | *Degree Centrality* | ***Eigenvector Centrality*** |
| --- | --- | --- | --- |
| MedStar Franklin Square | 3,522.2 | 301 | **1.000** |
| Johns Hopkins | 1,290.6 | 449 | **0.998** |
| Johns Hopkins Bayview | 3,153.3 | 415 | **0.893** |
| Lifebridge Sinai Hospital | 2,309.6 | 365 | **0.840** |
| University of Maryland | 379.4 | 442 | **0.584** |
| Mercy Medical Center | 2,283.7 | 391 | 0.493 |
| Greater Baltimore Medical Center | 2,245.7 | 308 | 0.455 |
| St. Agnes Hospital | 2,826.8 | 329 | 0.453 |
| MedStar Union Memorial | 4,156.7 | 366 | 0.411 |
| UM Saint Joseph | 3,528.6 | 344 | 0.410 |
| MedStar Good Samaritan | 3,086.6 | 280 | 0.359 |
| Lifebridge Northwest Hospital | 2,653.8 | 236 | 0.322 |
| UM Baltimore Washington Medical Center | 3,230.3 | 334 | 0.307 |
| UMM Center Midtown | 2,939.6 | 285 | 0.186 |
| Howard General Hospital | 1,987.1 | 295 | 0.181 |
| MedStar Harbor Hospital | 3,596.9 | 274 | 0.174 |
| Anne Arundel Medical Center | 2,726.5 | 389 | 0.167 |
| Bon Secours | 2,204.4 | 240 | 0.144 |
| Upper Chesapeake Medical Center | 4,391.6 | 199 | 0.119 |
| Meritus Health System (Wash. Co.) | 4,405.0 | 205 | 0.119 |
| Holy Cross Hospital | 2,695.5 | 317 | 0.090 |
| Carroll County General | 4,337.5 | 253 | 0.071 |
| UM Rehab & Orthopaedic Institute | 2,838.1 | 348 | 0.064 |
| Frederick Memorial | 5,349.3 | 263 | 0.050 |
| Shady Grove Adventist | 3,065.8 | 253 | 0.040 |
| Peninsula Regional | 4,941.5 | 282 | 0.037 |
| Harford Memorial Hospital | 4,030.0 | 187 | 0.036 |
| Lifebridge Levindale | 3,925.1 | 248 | 0.036 |
| Suburban Hospital | 3,727.1 | 298 | 0.030 |
| Prince George’s | 2,102.9 | 248 | 0.028 |
| MedStar Montgomery General | 2,579.3 | 259 | 0.027 |
| Western MD Health System | 6,193.6 | 166 | 0.024 |
| MedStar Southern Maryland | 1,702.0 | 241 | 0.024 |
| Washington Adventist | 2,455.8 | 269 | 0.024 |
| Greater Laurel | 2,415.7 | 256 | 0.023 |
| Doctors Community Hospital | 2,183.5 | 213 | 0.022 |
| UM Shore Medical Center at Easton | 5,124.9 | 194 | 0.015 |
| Mount Washington Pediatric Hospital | 5,049.8 | 232 | 0.015 |
| Union of Cecil | 4,635.0 | 176 | 0.013 |
| UM Charles Regional Medical Center | 3,391.2 | 146 | 0.008 |
| Atlantic General | 5,424.9 | 228 | 0.008 |
| MedStar Saint Mary’s Hospital | 2,946.0 | 184 | 0.008 |
| Calvert Memorial | 3,851.4 | 193 | 0.008 |
| UM Shore Medical Center at Dorchester | 3,992.9 | 184 | 0.006 |
| Holy Cross Hospital- Germantown | 2,383.9 | 166 | 0.006 |
| Fort Washington | 1,594.8 | 83 | 0.003 |
| Adventist Rehabilitation Hospital | 1,723.9 | 145 | 0.002 |
| UM Shore Medical Center Chestertown | 952.8 | 71 | 0.002 |
| Healthsouth Chesapeake Rehab Hospital | 2,277.0 | 85 | 0.001 |
| Garrett County | 3,089.0 | 80 | 0.001 |
| McCready | 17.1 | 17 | 0.000 |

# Economic evaluation

To evaluate the economic impact of implementing an electronic registry and varying rates of culture-based screening for CRE, we calculated the cost-effectiveness of each scenario as the cost required to avert one episode of HAI from the hospital perspective. The number of HAI episodes averted by each scenario was determined by the model. Cost inputs for each intervention scenario (Table 6) included the cost of implementing an electronic registry that would identify patients with CRE on admissions (applicable to scenarios 4-7 only), the cost of conducting active surveillance screening, and the cost of implementing a bundled IPC intervention (e.g., contact precautions and chlorhexidine bathing).

## Cost calculations

Where possible, unit costs were estimated based on literature and expert knowledge and multiplied by the number of unit quantities expected in each scenario as determined by the model. To determine the cost averted per HAI, we estimated the mean attributable cost of CRE infection from literature, which was found to be approximately $30,484 (95% CI, 28,437-32,530) per infection ^16-18^.

For scenarios 4-7, the estimated cost of implementing an electronic registry was roughly $10,000 per hospital facility ^19^. This cost was predominately based on the cost of staff salary required to update a facility’s electronic health record system so that patients with prior colonization or infection with CRE are flagged in the system.

The cost of active surveillance screening was broken down into four processes for which mean costs were estimated from literature and expert knowledge: (i) swabbing, which assumed one swab per body site, (ii) culturing, which included the material costs for MacConkey or chromogenic agar, ertapenem or meropenem disks, and tryptic soy broth, (iii) organism identification (e.g. matrix-assisted laser-desorption ionization time-of-flight mass spectrometry) and antimicrobial susceptibility testing (e.g., VITEK 2), and (iv) phenotypic testing (e.g., phenotypic modified carbapenem inactivation method) or molecular analysis (polymerase chain reaction test). We estimated these mean costs per patient screening to be $1.00 for swabbing ^20^, $4.09 for culturing ^20-22^ , $5.70 for organism identification test and antimicrobial susceptibility testing ^21^, and $37.04 for phenotypic testing or molecular analysis ^20-22^. Assuming 30% of cultures are positive and require organism identification and antimicrobial susceptibility testing, and 1-5% of cultures are CRO positive and require phenotypic screening or molecular analysis, we estimated the total cost per active surveillance screening test to be approximately $8.65. To determine the cost of implementing active surveillance screening for each scenario, we multiplied the cost per screening test by the mean number of patients screened per year in each scenario.

The cost of implementing a bundled IPC intervention for patients testing positive for CRE was estimated from literature and included the cost of placing a patient on contact precautions and the cost of implementing daily chlorhexidine (CHG) bathing for decolonization. Contact precautions was defined as the use of personal protective equipment, including disposable gloves and gowns, which require one-minute to don and doff per contact. The combined material costs ($1.11) and staffing costs ($0.56) for implementing contact precautions was $1.67 per healthcare worker-patient contact ^20^. Assuming an average of 35 contacts per day, we estimated contact precautions to cost approximately $58.33 per patient per day of hospitalization. As for the cost of daily CHG bathing, the mean cost per bath using CHG impregnated wipes was $5.62 ^23, 24^. Assuming a mean length of stay of 10 days ^18^, we estimated the cost of implementing the IPC bundle to be approximately $639.48 per CRE patient, which was then multiplied by the mean number of new positive detections per year to obtain total costs for each scenario.

| Table S6. Costs of intervention scenarios in USD | | | | | | |  |
| --- | --- | --- | --- | --- | --- | --- | --- |
| *Scenario* | *Number of acute hospitals receiving intervention* | *Mean number of patients screened per year* | *Mean number of new positive detections per year* | *Cost of screening (US$)* | *Cost of EHR intervention (US$)* | *Cost of bundled IPC (US$)* | |
| Baseline | 1 | 1,298 | 323 | 12,000 | 0 | 207,000 | |
| Scenario 1 | 5 | 10,141 | 442 | 88,000 | 0 | 283,000 | |
| Scenario 2 | 46 | 27,986 | 868 | 243,000 | 0 | 556,000 | |
| Scenario 3 | 46 | 19,567 | 3,267 | 170,000 | 0 | 2,090,000 | |
| Scenario 4 | 46 | 1,214 | 317 | 11,000 | 460,000 | 203,000 | |
| Scenario 5 | 46 | 9,474 | 405 | 82,000 | 460,000 | 260,000 | |
| Scenario 6 | 46 | 26,365 | 786 | 229,000 | 460,000 | 503,000 | |
| Scenario 7 | 46 | 18,232 | 2,931 | 158,000 | 460,000 | 1,875,000 | |

## Cost-effective analysis

The cost-effective analysis was conducted to compare the relative costs of implementing an electronic registry (along with increased rates of culture-based screening for CRE) to their modeled health outcome, an expected reduction in CRE infection. Net costs were calculated by subtracting the cost averted from reductions in CRE infection from the cost of interventions (Table 7). To summarize the cost-effectiveness of the various interventions scenarios, we measured the incremental cost-effectiveness ratio (ICER), calculated as the difference in cost between two interventions (e.g., scenario 4 and scenario 5), divided by the difference in their effect (i.e., an expected reduction in CRE infection).

| Table S7. Incremental cost-effectiveness ratio (ICER) of modeled interventions | | | |
| --- | --- | --- | --- |
| Scenario | *Mean infections averted per year compared to baseline* | *Annual net cost* | *ICER* |
| Baseline | *NA* | 218,000 | *NA* |
| Scenario 1^*^ | -0.7 | 393,000 | -209,000 |
| Scenario 2^*^ | 0.4 | 786,000 | 1,539,000 |
| Scenario 3^*^ | 3.7 | 2,146,000 | 552,000 |
| Scenario 4^*^ | 18.8 | 101,000 | 25,000 |
| Scenario 5^†^ | 19.2 | 216,000 | 288,000 |
| Scenario 6^†^ | 19.8 | 588,000 | 499,000 |
| Scenario 7^†^ | 20.9 | 1,855,000 | 856,000 |
| * Incremental to baseline; ^†^ Incremental to Scenario 4 | | |  |
|  | | |  |

# Sensitivity Analysis

## Partial Rank Correlation Coefficient (PRCC) Analysis

Figure S4 shows the PRCC from the global sensitivity analysis of all sampled parameters using the Latin Hyper Cube sampling (LHS) algorithm. The partial rank correlation coefficient effectively measures the strength of a linear association between an input and an output. In our sensitivity analysis, the measured output is total colonization at acute care hospital which is a key variable in our cost-effectiveness analysis. This sensitivity analysis was conducted on the Baseline scenario. The analysis was done on 300 sampled points. The sampling ranges for each parameter are listed in Table S5 in Appendix F. We found that the most sensitive parameters were $\beta_{j}^{L}$,$\rho_{0}$, and $\psi$.

## Transmission Reduction of IPC Bundle

We conducted a sensitivity analysis around the parameter σ to simulate varying effectiveness around infection prevention and control (IPC) interventions within acute care facilities. Since most hospitals vary in resources, we varied the effectiveness around σ and show that detection, contact precautions, and daily chlorhexidine (CHG) bathing are needed to mitigate the spread of CRE to other patients, HCWs and visitors in acute care facilities. Figure S3 shows the reduction in infections, colonization, and deaths at acute care hospitals relative to different values of σ.


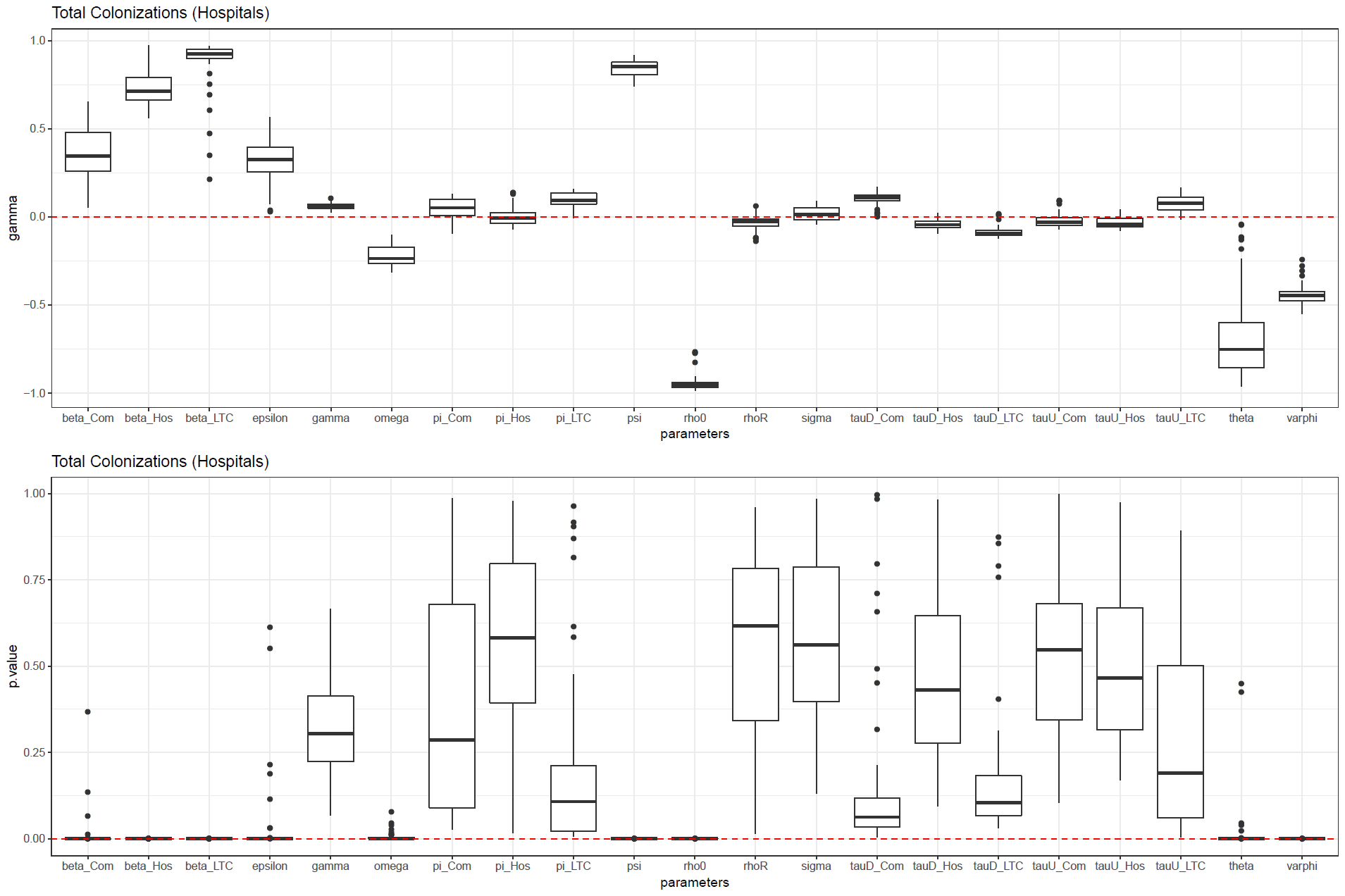


**Figure S3. PRCC Sensitivity Analysis Results.** (Top) box-and-whisker plot represents the calculated PRCC values (gamma) for each LHS sampled parameter. The box and whiskers represent the inner 50% percentile indicate 1.5 times the interquartile range. (Below) The P-values for the estimated PRCC for all parameters are shown in the box-whisker plot with the same convention as above.


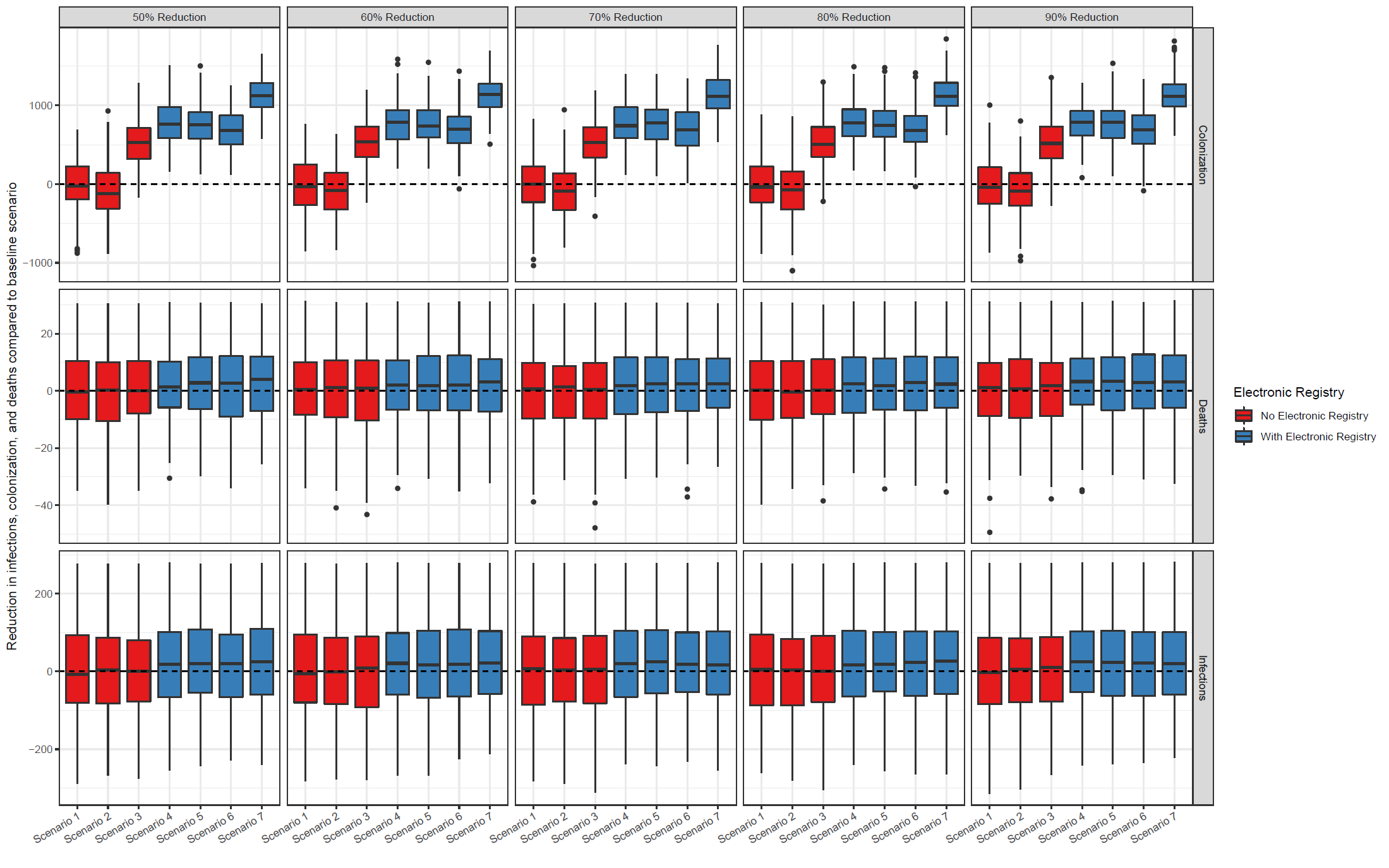


**Figure S4. IPC sensitivity analysis relative to reductions in colonization, infections, and deaths.** The sensitivity analysis varies the levels of reduction in transmission due to bundled IPC interventions. The box represents the inner 50% percentile, and whiskers indicate 1.5 times the interquartile range. We varied the parameter σ that corresponds to 50%, 60%, 70%, 80%, and 90% in reduction in transmission due to IPC.

**References**

1. Marchaim D, Chopra T, Bhargava A, et al. Recent exposure to antimicrobials and carbapenem-resistant Enterobacteriaceae: the role of antimicrobial stewardship*. Infection Control & Hospital Epidemiology*. 2012;33(8):817-830.

2. Schwaber MJ, Klarfeld-Lidji S, Navon-Venezia S, Schwartz D, Leavitt A, Carmeli Y. Predictors of carbapenem-resistant Klebsiella pneumoniae acquisition among hospitalized adults and effect of acquisition on mortality*. Antimicrob Agents Chemother*. 2008;52(3):1028-1033.

3. Falagas ME, Rafailidis PI, Kofteridis D, et al. Risk factors of carbapenem-resistant Klebsiella pneumoniae infections: a matched case–control study*. J Antimicrob Chemother*. 2007;60(5):1124-1130.

4. van Loon K, Voor in ‘t holt, Anne F, Vos MC. A Systematic Review and Meta-analyses of the Clinical Epidemiology of Carbapenem-Resistant Enterobacteriaceae*. Antimicrobial agents and chemotherapy*. 2017;62(1). doi: 10.1128/aac.01730-17.

5. Borer A, Eskira S, Nativ R, et al. A multifaceted intervention strategy for eradication of a hospital-wide outbreak caused by carbapenem-resistant Klebsiella pneumoniae in Southern Israel*. Infection Control & Hospital Epidemiology*. 2011;32(12):1158-1165.

6. Lin MY, Lolans K, Blom DW, et al. The effectiveness of routine daily chlorhexidine gluconate bathing in reducing Klebsiella pneumoniae carbapenemase–producing Enterobacteriaceae skin burden among long-term acute care hospital patients*. Infection Control & Hospital Epidemiology*. 2014;35(4):440-442.

7. Klein E. Antibiotics treatment at Johns Hopkins. 2019.

8. Bar-Yoseph H, Hussein K, Braun E, Paul M. Natural history and decolonization strategies for ESBL/carbapenem-resistant Enterobacteriaceae carriage: systematic review and meta-analysis*. J Antimicrob Chemother*. 2016;71(10):2729-2739.

9. Kouyos R, Klein E, Grenfell B. Hospital-community interactions foster coexistence between methicillin-resistant strains of Staphylococcus aureus*. PLoS pathogens*. 2013;9(2).

10. Satlin MJ, Kubin CJ, Blumenthal JS, et al. Comparative effectiveness of aminoglycosides, polymyxin B, and tigecycline for clearance of carbapenem-resistant Klebsiella pneumoniae from urine*. Antimicrob Agents Chemother*. 2011;55(12):5893-5899.

11. Kang C, Kim S, Kim DM, et al. Risk factors for and clinical outcomes of bloodstream infections caused by extended-spectrum beta-lactamase-producing Klebsiella pneumoniae*. Infection Control & Hospital Epidemiology*. 2004;25(10):860-867.

12. Gallagher JC, Kuriakose S, Haynes K, Axelrod P. Case-case-control study of patients with carbapenem-resistant and third-generation-cephalosporin-resistant Klebsiella pneumoniae bloodstream infections*. Antimicrob Agents Chemother*. 2014;58(10):5732-5735.

13. Kritsotakis EI, Tsioutis C, Roumbelaki M, Christidou A, Gikas A. Antibiotic use and the risk of carbapenem-resistant extended-spectrum-β-lactamase-producing Klebsiella pneumoniae infection in hospitalized patients: results of a double case–control study*. J Antimicrob Chemother*. 2011;66(6):1383-1391.

14. McConville TH, Sullivan SB, Gomez-Simmonds A, Whittier S, Uhlemann A. Carbapenem-resistant Enterobacteriaceae colonization (CRE) and subsequent risk of infection and 90-day mortality in critically ill patients, an observational study*. PLoS One*. 2017;12(10).

15. Bonacich P. Factoring and weighting approaches to status scores and clique identification*. Journal of mathematical sociology*. 1972;2(1):113-120.

16. Cosgrove SE, Kaye KS, Eliopoulous GM, Carmeli Y. Health and economic outcomes of the emergence of third-generation cephalosporin resistance in Enterobacter species*. Arch Intern Med*. 2002;162(2):185-190.

17. Almario CV, May FP, Shaheen NJ, et al. Cost utility of competing strategies to prevent endoscopic transmission of carbapenem-resistant enterobacteriaceae*. Am J Gastroenterol*. 2015;110(12):1666.

18. Bartsch SM, McKinnell JA, Mueller LE, et al. Potential economic burden of carbapenem-resistant Enterobacteriaceae (CRE) in the United States*. Clinical Microbiology and Infection*. 2017;23(1):48. e9-48. e16.

19. Levin S. Electronic Health Registry Cost. 2019.

20. McKinnell JA, Bartsch SM, Lee BY, Huang SS, Miller LG. Cost-benefit analysis from the hospital perspective of universal active screening followed by contact precautions for methicillin-resistant Staphylococcus aureus carriers*. infection control & hospital epidemiology*. 2015;36(1):2-13.

21. Mathers AJ, Poulter M, Dirks D, Carroll J, Sifri CD, Hazen KC. Clinical microbiology costs for methods of active surveillance for Klebsiella pneumoniae carbapenemase–producing Enterobacteriaceae*. Infection Control & Hospital Epidemiology*. 2014;35(4):350-355.

22. Simner P. Testing and Screening Costs. 2019.

23. Reagan KA, Chan DM, Vanhoozer G, et al. You get back what you give: Decreased hospital infections with improvement in CHG bathing, a mathematical modeling and cost analysis*. Am J Infect Control*. 2019;47(12):1471-1473.

24. Petlin A, Schallom M, Prentice D, et al. Chlorhexidine gluconate bathing to reduce methicillin-resistant Staphylococcus aureus acquisition*. Crit Care Nurse*. 2014;34(5):17-24.
